# Structure analysis and antiviral activity of CW-33 analogues against Japanese encephalitis virus

**DOI:** 10.1038/s41598-018-34932-4

**Published:** 2018-11-09

**Authors:** Jin-Cherng Lien, Ching-Ying Wang, Hsueh–Chou Lai, Chien-Yi Lu, Yu-Fong Lin, Ging-Yan Gao, Kuan-Chung Chen, An-Cheng Huang, Su-Hua Huang, Cheng-Wen Lin

**Affiliations:** 1 School of Pharmacy, China Medical University, Taichung, Taiwan; 2 Department of Medical Laboratory Science and Biotechnology, China Medical University, Taichung, Taiwan; 3 School of Chinese Medicine, China Medical University, Taichung, Taiwan; 4 Division of Hepato-gastroenterology, department of internal medicine, China Medical University Hospital, Taichung, Taiwan; 5 Department of Nursing, St. Mary’s Junior College of Medicine, Nursing and Management, Yilan County, Taiwan; 6 Department of Biotechnology, Asia University, Wufeng, Taichung Taiwan; 7 Chinese Medicine Research center, China Medical University, Taichung, Taiwan

## Abstract

Japanese encephalitis virus (JEV) is a member of neurotropic flaviviruses transmitted by mosquito bites, causing severe central nervous system disorders. Current JEV genotype III vaccines have a low protection against genotype I isolates in the risk zone. The lead compound CW-33, ethyl 2-(3′,5′-dimethylanilino)-4-oxo-4,5-dihydrofuran-3-carboxylate, demonstrates the antiviral activity against JEV with an IC_50_ values of 38.5 μM for virus yield reduction (Int J Mol Sci 2016,17: E1386). This study synthesized fourteen CW-33 analogues containing a fluoro atom or one methoxy group at the C-2, C-3, or C-4 of anilino ring, and then evaluated for their antiviral activity and mechanism. Among 6 amalogues, CW-33A (ethyl 2-(2-fluoroanilino)-4-oxo- 4,5-dihydrofuran-3-carboxylate), and CW-33D (ethyl 2-(3-methoxyanilino)-4-oxo- 4,5-dihydrofuran-3-carboxylate exhibited antiviral potentials in viral cytopathic effect (CPE) inhibition. CW-33A significantly suppressed the viral protein expression, genome synthesis and intracellular JEV particle production, showing a higher inhibitory effect on JEV yield than CW-33 and CW-33D. The study demonstrated that a mono-fluoro substitution on at the C-2 anilino ring of CW-33 improved the antiviral activity JEV, revealing the structure-activity relationship for developing novel agents against JEV infection.

## Introduction

Japanese encephalitis virus (JEV) is a neurotropic flavivirus that causes acute flaccid paralysis, aseptic meningitis, and encephalitis^1,2^. Like other pathogenic flaviviruses (dengue virus, West Nile virus, and Zika virus), JEV is an enveloped infectious particle with a positive-sense single-stranded RNA genome that encodes a single large polyprotein precursor of structural proteins (capsid, pre-membrane and envelope proteins) and non-structural proteins (NS1, NS2A, NS2B, NS3, NS4A, NS4B and NS5 proteins)^1,2^. JEV spread is transmitted by mosquitoes in Asia and Australia, threatening public health of over 3 billion people in this risk zone^3^. JEV infection brings about greater than 30,000 JE cases per year included near 10,000 deaths. Meanwhile, a half of JE survivors show motor neuron and cranial nerve symptoms, epilepsy, and mental disorders^3^. Recent, inactivated Nakayama and attenuated SA14-14-2 JEV vaccines have been demonstrated to reduce the protection in children from the JEV infection because JEV genotype I isolates in the risk zone were resistant to genotype III vaccine-induced neutralizing antibodies^4,5^. Importantly, the prevalence of JEV genotype I strain has been increasing in recent years^6–8^.

**Figure 1:**
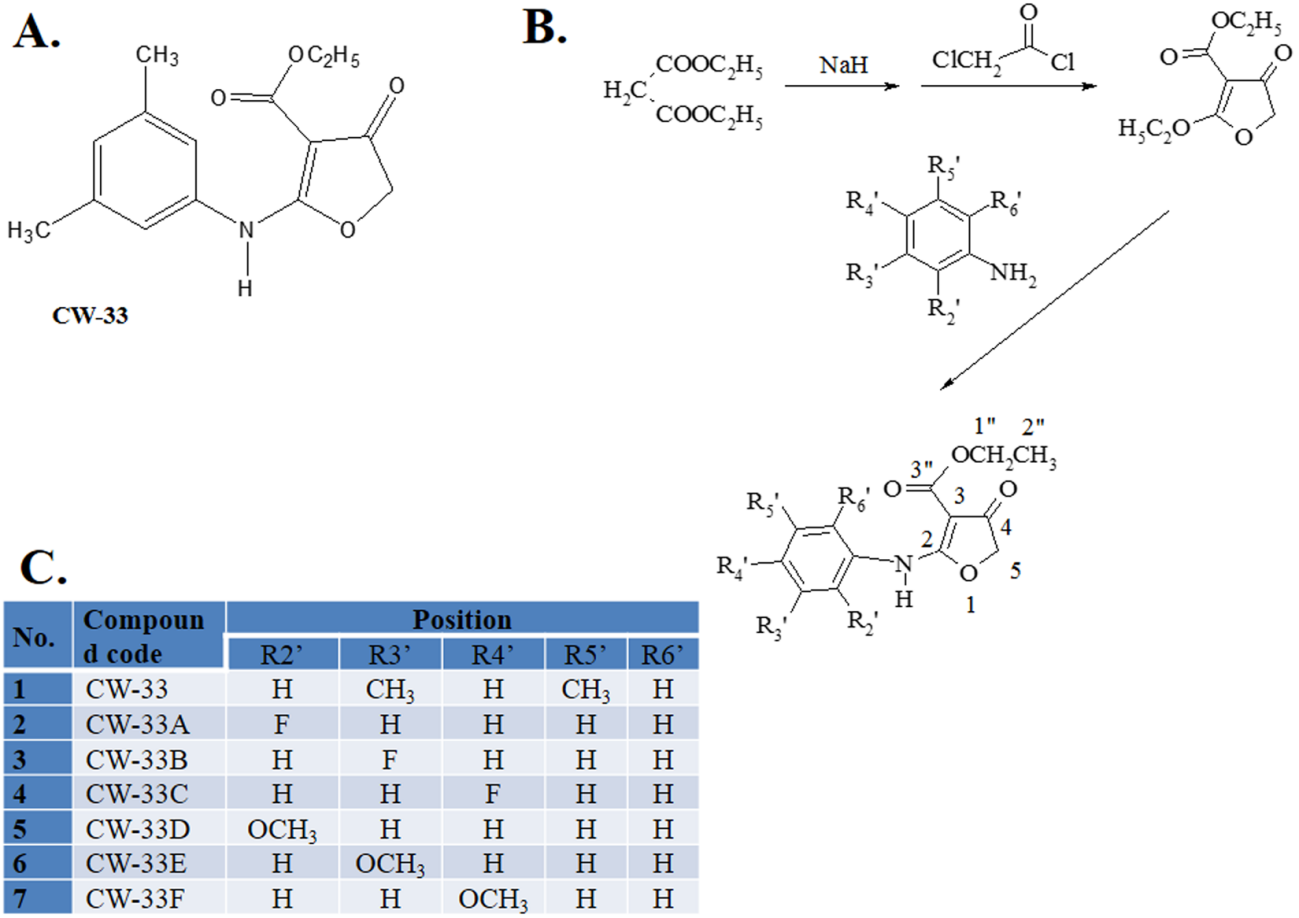
Structure of compound CW-33 (**A**), synthesis of CW-33 analogues (**B**) and mono-substituted groups at C-2, C-3 and C-4 position in aniline ring of CW-33 analogues (**C**).

The compound CW-33, ethyl 2-(3′,5′-dimethylanilino)-4-oxo-4,5- dihydrofuran-3-carboxylate (Fig. 1A), exhibits antiviral activity against JEV^9^. The half maximal inhibitory concentration (IC_50_) of CW-33 to reduce the progeny virus production *in vitro* is greater than 10 μM, thus the modification of CW-33 structure to improve antiviral activity is essential. To date, the mono substituent at C-2, C-3, or C-4 of the aniline ring in CW-33 was synthesized (Fig. 1), and characterized by 1 H NMR and 13 C NMR (Table 1). The analogues of CW-33 were named as CW-33A, CW-33B, CW-33C were divided into five classes according to the modification: fluoro- (CW-33A, CW-33B, CW-33C) and methoxy- (CW-33D, CW-33E, CW-33F) analogues (Table 1). Herein, anti-JEV activity of these CW-33 analogues was further investigated, such as cytopathic effect (CPE) inhibition and virus yield reduction assays. Moreover, the structure-activity relationship and antiviral mechanism(s) of bioactive CW-33 analogues were examined using the viral translation and replication assays with JEV replicon, and the viral titer assay of intracellular viral particles.

**Table 1:** Structure and NMR spectrum of CW-33 derivatives.

Compound code	Structure	Yield (%)	M.P. (°C)	1H NMR (200 MHz, CDCl_3_)	13C NMR (50 MHz, CDCl_3_)
**CW-33A**		54.26	140–142	δ1.38 (3 H, *t*, *J* = 7.2 Hz, H-2″), 4.38 (2 H, *q*, *J* = 7.2 Hz, H-1″), 4.68 (2 H, *s*, H-5), 7.14–7.22 (3 H, *m*, H-4′, H-5′, H-6′), 7.69–7.74 (1 H, *m*, H-3′), 10.44 (1 H, *s*, NH)	δ14.25 (C-2″), 60.49 (C-1″), 75.39 (C-5), 88.16 (C-3), 115.56, 115.94 (C-6′), 122.44 (C-3′), 123.15, 123.37 (C-1′), 124.37, 124.51 (C-4′), 126.60, 126.75 (C-5′), 151.01, 155.94 (C-2′), 165.08 (C-2), 177.82 (C-3″), 188.24 (C-4)
**CW-33B**		53.4	136-137	δ1.39 (3 H, *t*, *J* = 7.2 Hz, H-2″), 4.38 (2 H, *q*, *J* = 7.2 Hz, H-1″), 4.72 (2 H, *s*, H-5), 6.92–7.12 (2 H, *m*, H-4′, H-6′), 7.20–7.23 (1 H, *m*, H-5′), 7.32–7.38 (1 H, *m*, H-2′), 10.38 (1 H, *s*, NH)	δ14.46 (C-2″), 60.78 (C-1″), 75.60 (C-5), 87.99 (C-3), 108.61, 108.87 (C-2′), 112.81, 113.02 (C-4′), 116.73 (C-6′), 130.72, 130.81 (C-5′), 136.21, 136.32 (C-1′), 161.74, 164.20 (C-3′), 165.58 (C-2), 177.81 (C-3″), 188.30 (C-4)
**CW-33C**		44.49	166-167	δ1.32 (3 H, *t*, *J* = 7.0 Hz, H-2″), 4.30 (2 H, *q*, *J* = 7.0 Hz, H-1″), 4.62 (2 H, *s*, H-5), 7.02–7.06 (2 H, *m*, H-2′, H-6′), 7.29–7.31 (2 H, *m*, H-3′, H-5′), 10.17 (1 H, *s*, NH)	δ14.36 (C-2″), 60.52 (C-1″), 75.34 (C-5), 87.49 (C-3), 116.11, 116.34 (C-2′, C-6′), 123.39, 123.48 (C-3′, C-5′), 130.63 (C-1′), 159.20, 161.65 (C-4′),165.45 (C-2), 177.55 (C-3″), 188.21 (C-4)
**CW-33D**		60.29	180-181	δ1.39 (3 H, *t*, *J* = 7.0 Hz, H-2″), 3.95 (3 H, *s*, C2′-OCH_3_), 4.38 (2 H, *q*, *J* = 7.0 Hz, H-1″), 4.69 (2 H, *s*, H-5), 6.93–7.00 (2 H, *m*, H-4′, H-6′), 7.12–7.26 (1 H, *m*, H-5′), 7.73 (1 H, *dd*, *J* = 8.3 Hz, 1.8 Hz, H-3′), 10.64 (1 H, *s*, NH)	δ14.31 (C-2″), 55.87 (C2′-OCH_3_), 60.20 (C-1″), 75.20 (C-5), 87.91 (C-3), 110.68 (C-4′), 120.46 (C-6′), 120.63 (C-3′), 124.57 (C-1′), 125.86 (C-5′), 149.20 (C-2′), 164.94 (C-2), 177.10 (C-3″), 188.38 (C-4)
**CW-33E**		41.11	139-140	δ1.39 (3 H, *t*, *J* = 7.1 Hz, H-2″), 3.82 (3 H, *s*, C3′-OCH_3_), 4.37 (2 H, *q*, *J* = 7.0 Hz, H-1″), 4.67 (2 H, *s*, H-5), 6.77 (1 H, *d*, *J* = 7.8 Hz, H-6′), 6.94–6.97 (2 H, *m*, H-2′, 4′), 7.29 (1 H, *dd*, *J* = 7.8 Hz, H-6′), 10.26 (1 H, *s*, NH)	δ14.25 (C-2″), 55.21 (C3′-OCH_3_), 60.38 (C-1″), 75.25 (C-5), 87.48 (C-3), 107.29 (C-2′), 111.27 (C-4′), 113.38 (C-6′), 130.03 (C-5′), 135.65 (C-1′), 160.15 (C-3′), 165.39 (C-2), 177.48 (C-3″), 188.14 (C-4)
**CW-33F**		51.80	138-139	δ1.37 (3 H, *t*, *J* = 7.2 Hz, H-2″), 3.80 (3 H, *s*, C4′-OCH_3_), 4.35 (2 H, *q*, *J* = 7.0 Hz, H-1″), 4.63 (2 H, *s*, H-5), 6.89 (2 H, *d*, *J* = 6.8 Hz, H-2′, 6′), 7.27 (2 H, *d*, *J* = 6.8 Hz, H-3′, 5′), 10.08 (1 H, *s*, NH)	δ14.29 (C-2″), 55.27 (C4′-OCH_3_), 60.29 (C-1″), 75.12 (C-5), 87.09 (C-3), 114.34 (C-2′, C-6′), 123.22 (C-3′, C-5′), 127.28 (C-1′), 157.69 (C-4′), 165.37 (C-2), 177.22 (C-3″), 188.13 (C-4)

## Results

### Synthesis and characterization of CW-33 analogues containing the C-2, C-3, or C-4 anilino substituents

To improve anti-JEV activity, altering R2′, R3′, and R4′ substituents of CW-33, 2-(3′,5′-dimethylanilino)- 4-oxo-4,5-dihydrofuran-3-carboxylate (Compound 1, Fig. 1A) were generated, respectively. Placing an F atom at 2′-, 3′-, or 4′-position yielded CW-33A, CW-33B, and CW-33C (compounds 2–4). Substitution of 2′-, 3′-, or 4′-position with OCH_3_ (CW-33D, CW-33E, and CW-33F (compounds 5–7)) was also synthesized according to the scheme shown in Fig. 1B. The CW-33 analogues were purified using high-performance liquid chromatography, and then their identity and purity were examined using nuclear magnetic resonance (NMR) spectroscopy and mass spectrum (Fig. 1C, Supplemental Figs 1–12, Table 1).

#### Structure-activity relationships of CW-33 analogues

**Figure 2:**
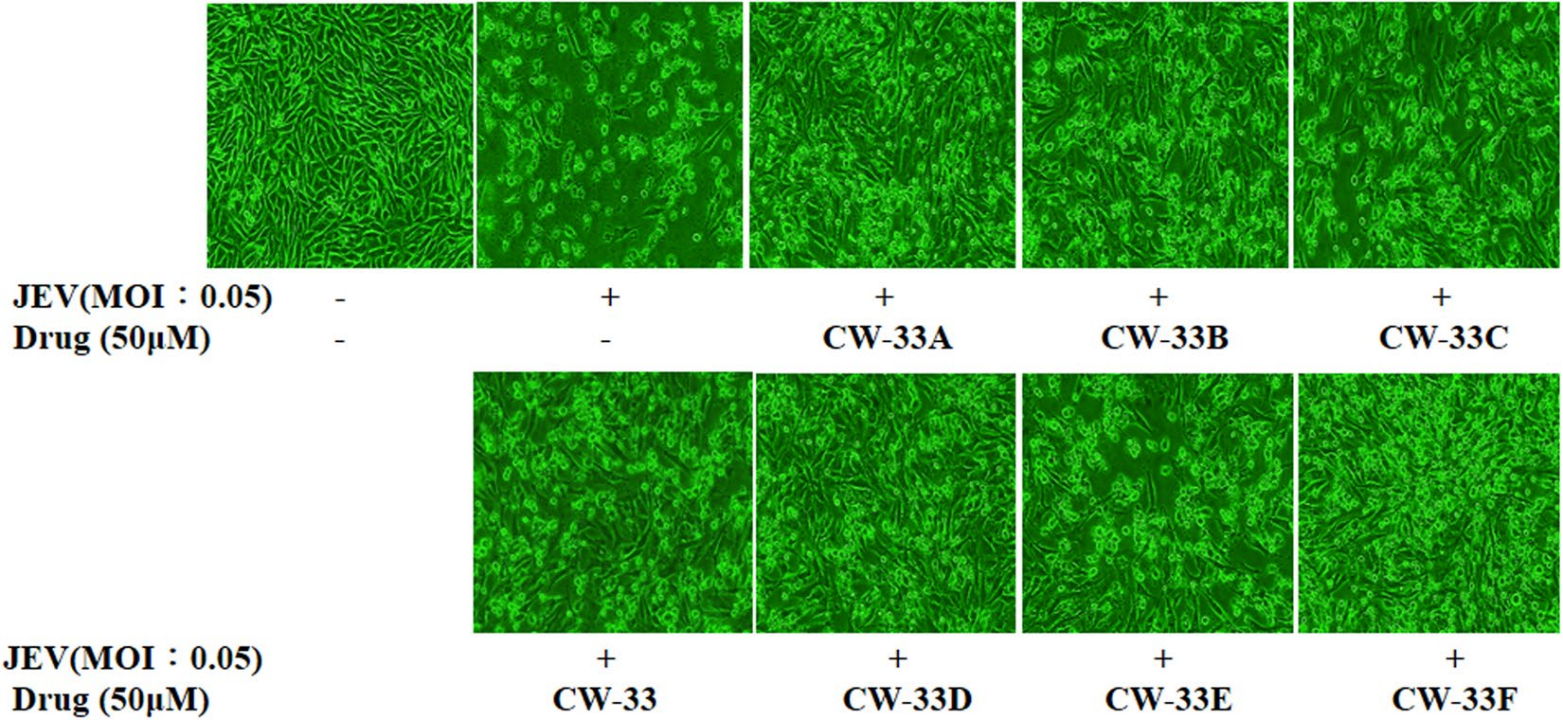
Inhibition of JEV-induced cytopathic effects by CW-33 analogues. TE-671 cells were infected with JEV at a MOI of 0.05 and immediately treated with CW-33 analogues. Images of JEV-induced cytopathic effect were photographed 36 hpi by phase-contrast microscopy.

**Figure 3:**
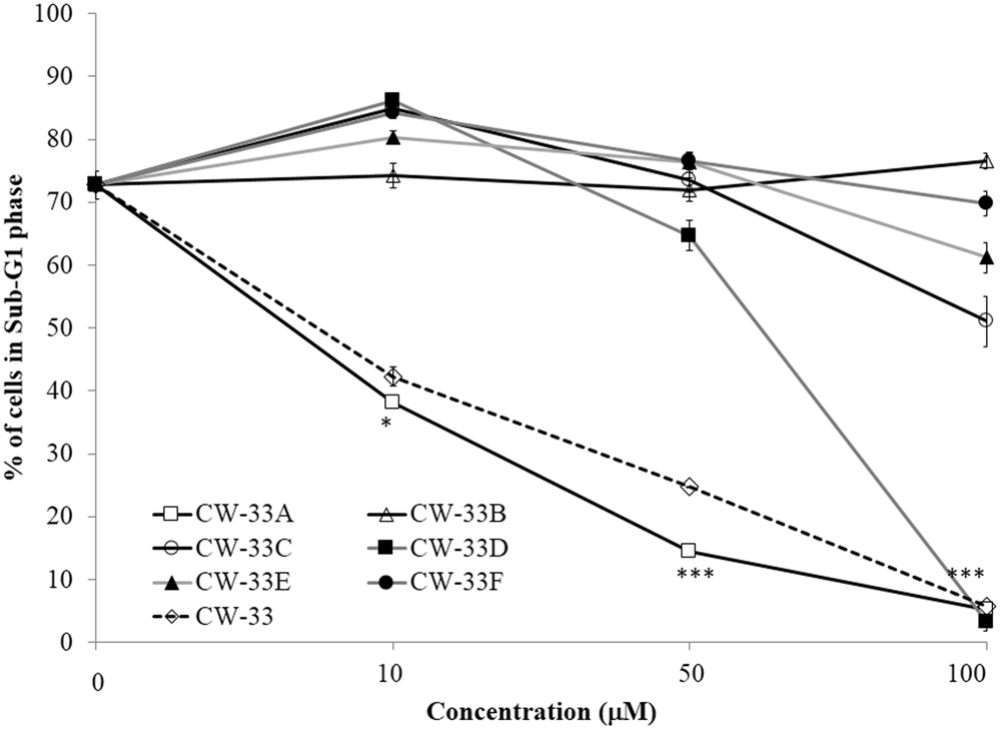
Sub-G1 reduction in JEV-infected cells by CW-33 analogues. Infected cells treated with or without CW-33 analogues were harvested 36 hpi, stained by PI dye, and then analyzed using flow cytometry. *p value < 0.05; ***p value < 0.001 compared with untreated cells.

To identify the relationship between structure and antiviral activity of CW-33 analogues against Japanese encephalitis virus, these compounds were tested their inhibitory effect on viral cytopathic effect (CPE) and apoptotic induction of TE-671cells infected JEV at an MOI of 0.05 (Figs 2 and 3). Cells were infected with JEV and simultaneously added with different concentrations of the analogues (10, 50 and 100 μM). Microscopic photography indicated CW-33A and CW-33D at 50 μM significantly lessening viral CPE 36 hours post infection (hpi), respectively (Fig. 2). The 50% inhibitory concentration (IC_50_) values is tested from the cell cycle analysis of treated infected cells using flow cytometry with PI staining showed that CW-33, CW-33A – CW-33F were 2.1, 1.1, 2538.1, 205.7, 24.9, 446.4 and 1612.9 μM, respectively (Fig. 3, Supplemental Fig. 13). Some of them showed the significant antiviral activity; the compound CW-33A had the potent activity with the IC50 value of near 1 μM. Taken together, these results suggest that the derivatives with a core structure of 2-anilino-4,5-dihydrofuran-3-carboxylate significantly inhibited Japanese encephalitis virus proliferation. Further aspects the structure and activity relationships of these compounds, it found that the positional isomers of the F or OCH_3_ substituent at the ortho, meta, or para position of the aniline ring showed the same activities trend that is ortho isomer > para isomer > meta isomer, i.e. CW-33A > CW-33C > CW-33B and CW-33D > CW-33F > CW-33E.

**Figure 4:**
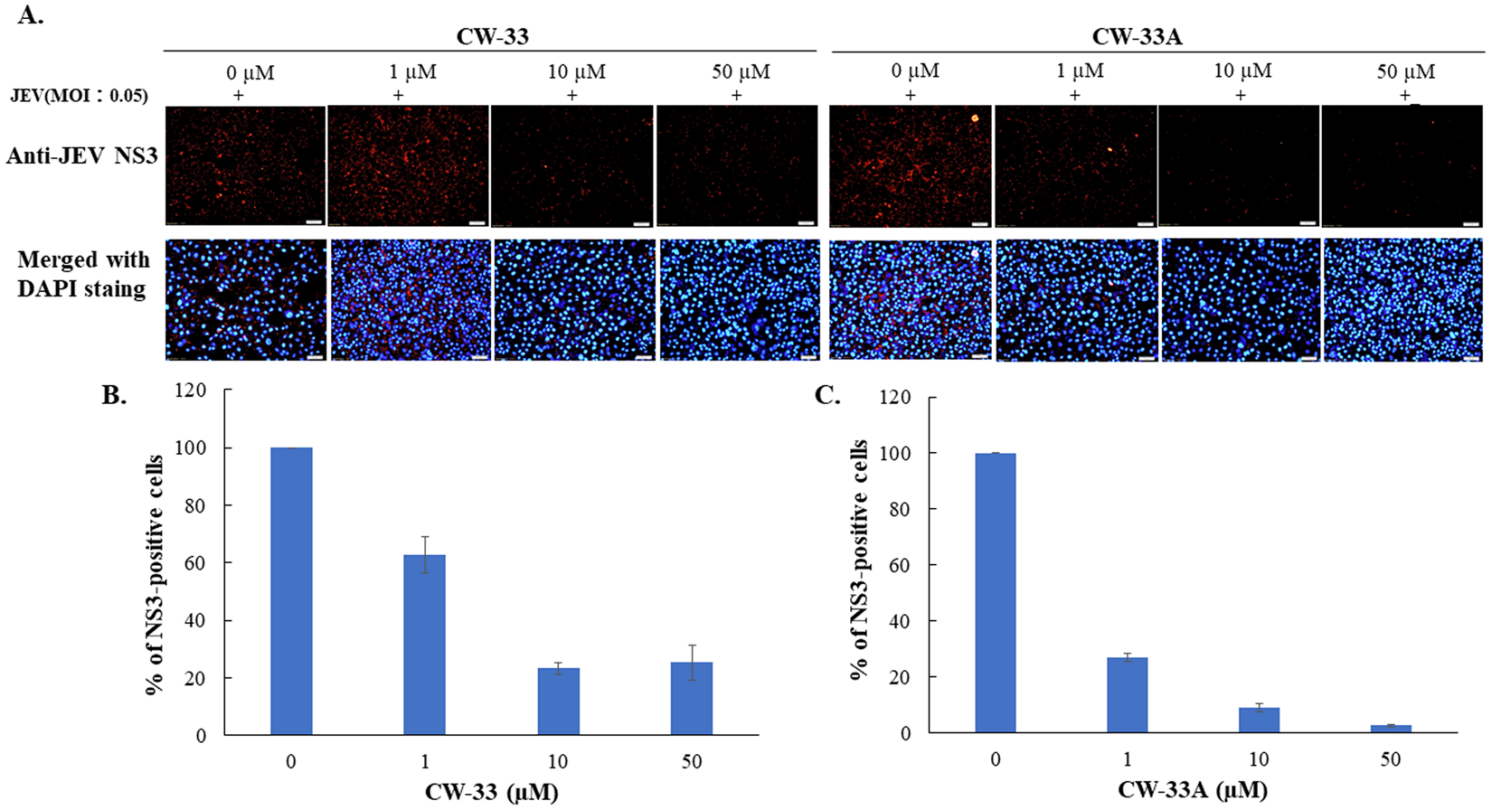
Inhibition of JEV infection by CW-33 and CW-33A during a 24-h incubation. JEV-infected cells with an MOI of 0.05 were performed using immunofluorescence staining after a 24-h treatment with CW-33 and CW-33A. JEV-infected cells were discovered with anti-NS3 and secondary antibodies; total cells were stained with DAPI (**A**). Infectivity was determined according to the ratio of NS3-positive cells to total cells (**B**,**C**).

When compared CW-33A, CW-33B, CW-33C with CW-33D, CW-33E, CW-33F, respectively, the compounds with electron withdrawing group F substituent was more activity than the compounds electron donating group OCH_3_ substituent. The compounds CW-33A and CW-33D established the best anti-JEV activity and dose-dependently diminished the sub-G1 phase of infected cells. Among them, CW-33A, a mono-fluoro substitution on at the meta position of the aniline ring, had a higher inhibitory activity on the apoptotic fraction of JEV-infected cells (IC_50_ = 1.1 ± 0.1 μM) than CW-33 (IC_50_ = 2.1 ± 0.3 μM). In addition, infectivity inhibition assay with immunofluorescent staining was performed when JEV-infected cells with an MOI of 0.05 were treated with the indicated concentrations of CW-33 and CW-33A after a 24-h incubation. Infectivity was determined according to the ratio of NS3-positive cells to total cells stained with DAPI (4′,6-diamidino-2-phenylindole). CW-33A and CW-33 concentration-dependently reduced the percentage of infected cells, however CW-33A had a higher inhibitory effect on infectivity during a 24 h period post JEV infection *in vitro* (IC_50_ = 0.2 ± 0.1 μM) than CW-33 (IC_50_ = 2.1 ± 0.1 μM) (Fig. 4).

#### Antiviral activity of CW-33A against JEV

**Figure 5:**
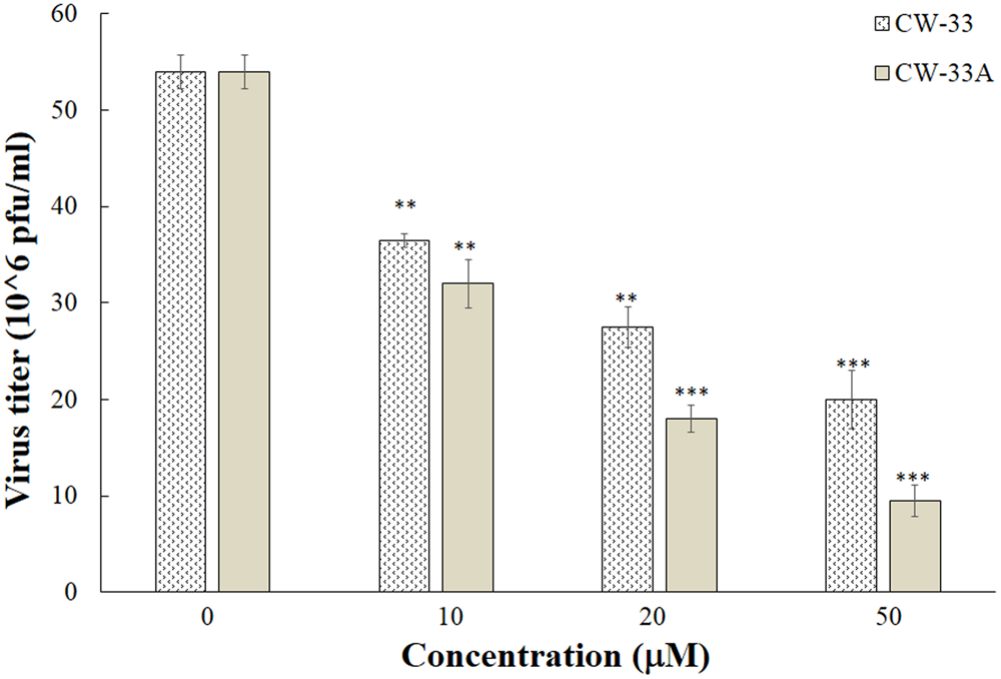
Reduction of JEV yield in human medulloblastoma cells by CW-33A. Supernatant of treated infected cells with CW-33 and CW-33A was harvested 36 hpi for measuring virus yield by plaque assay. **p value < 0.01; ***p value < 0.001 compared with mock-treated cells.

**Figure 6:**
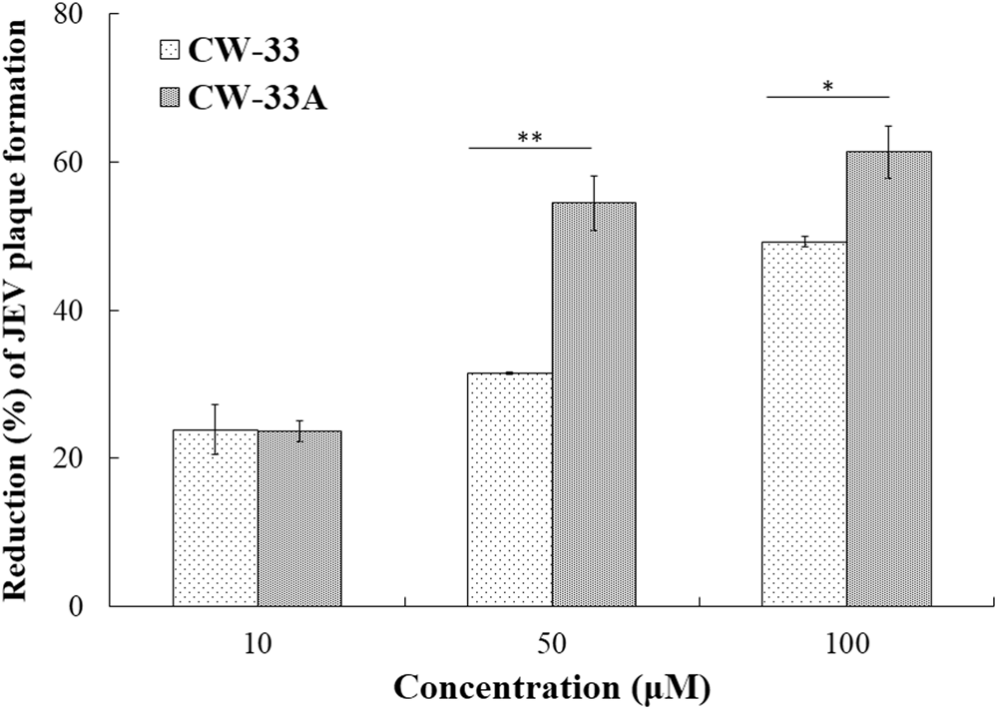
Quantitative reduction of JEV plaque formation by CW-33A using plaque assay. BHK-21 cells were co-treated with 100 pfu JEV and CW-33 or CW-33A for 1-h, overlaid with 1.1% methylcellulose-containing MEM medium for 72 h, and then dyed by naphthol blue-black. Finally, JEV plaque number was counted. *p value < 0.05; **p value < 0.01.

To further examine whether CW-33A has better inhibitory effect on the *in vitro* production of progeny viruses and plaque formation, the virus yield in cultured media and plaque formation of infected and treated cells was determined using plaque assay, respectively (Figs 5 and 6, Table 2). Quantitative analysis of virus yields in each supernatant of treated infected cells demonstrated CW-33A concentration-dependently inhibiting the production of progeny JEV in TE-671 cells (Fig. 5). The IC50 value of CW-33A for virus yield reduction (IC_50_ = 11.4 ± 2.2 μM) was lower than CW-33 (IC_50_ = 21.6 ± 2.3 μM). Meanwhile. CW-33A significantly inhibited JEV plaque formation in BHK-21 cells (Fig. 6), in which IC_50_ values on reducing virus plaque formation were 42.6 ± 9.4 μM μM for CW-33A and 103.8 ± 5.4 μM for CW-33, respectively. Thus, CW-33A had a higher therapeutic index value than CW-33. The results of CPE inhibition, apoptotic reduction, virus yield reduction and plaque formation inhibition revealed that CW-33A exhibited the higher antiviral activity against JEV than CW-33.

**Table 2:** IC50 values of CW-33A and CW-33 in different anti-JEV assays.

Compound code	IC50 (μM)^a^			
	Sub-G1 reduction	Infectivity reduction	Virus yield inhibition	Plaque formation reduction
CW-33A	1.1 ± 0.2	0.2 ± 0.1	11.4 ± 2.2	42.6 ± 9.4
CW-33	2.1 ± 0.3	2.1 ± 0.1	21.6 ± 2.3	103.8 ± 5.4

^a^IC50, 50% inhibitory concentration.

#### CW-33A-mediated inhibition of intracellular infectious virus production, viral protein and genome synthesis

**Figure 7:**
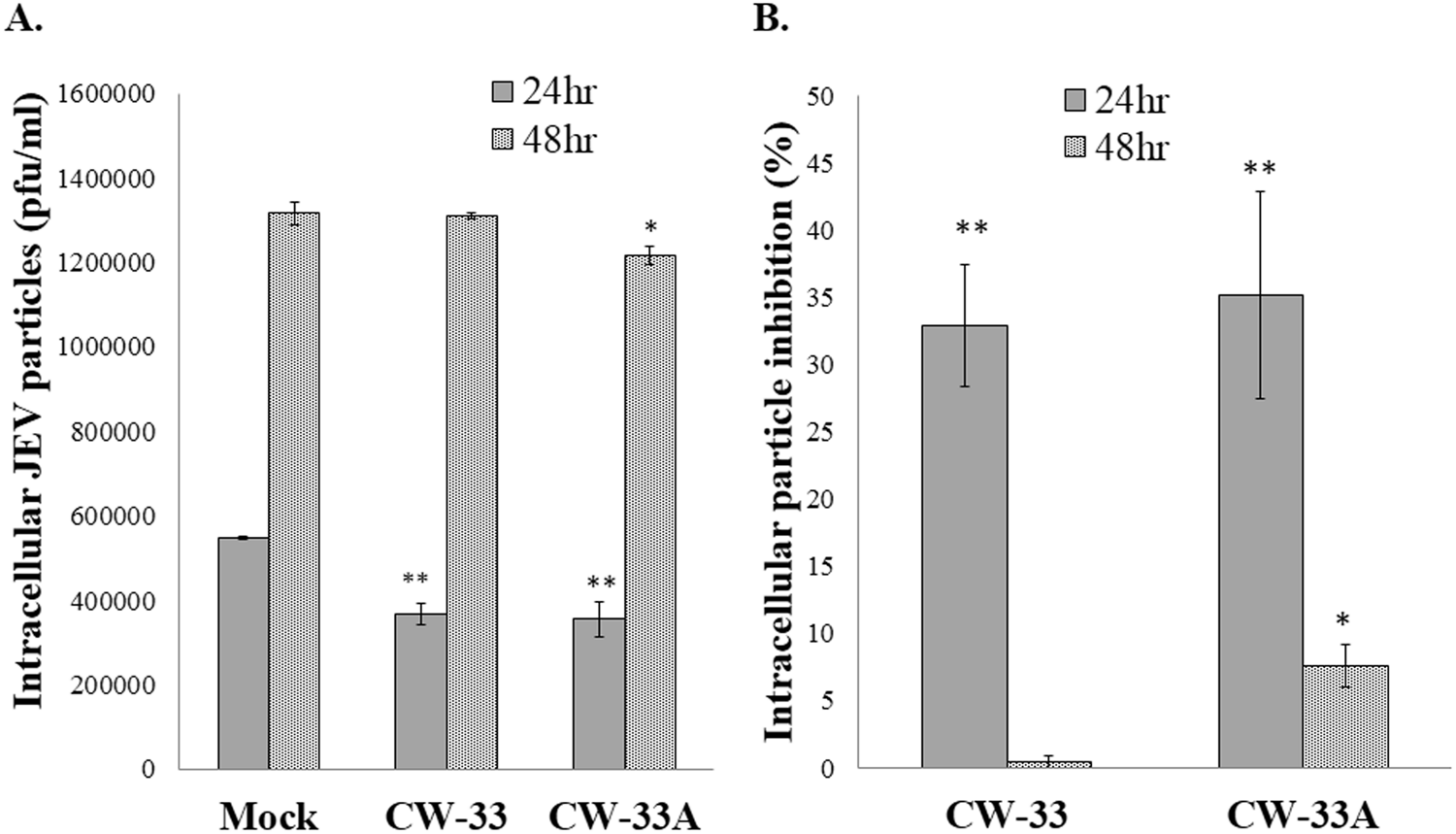
Suppression of intracellular virion production by CW-33A. Cells were infected with JEV, immediately treated with 10 μM of CW-33 and CW-33A, respectively, and then harvested 24 and 48 hpi. The treated and infected cells were lysed by three freeze-thaw cycles. The titer of intracellular infectious particles was determined by plaque assay (**A**). The inhibitory rate was determined according to the ration of loss particle number to mock-treated group (**B**). *p value < 0.05; **p value < 0.01. compared with mock-treated infected cells.

**Figure 8:**
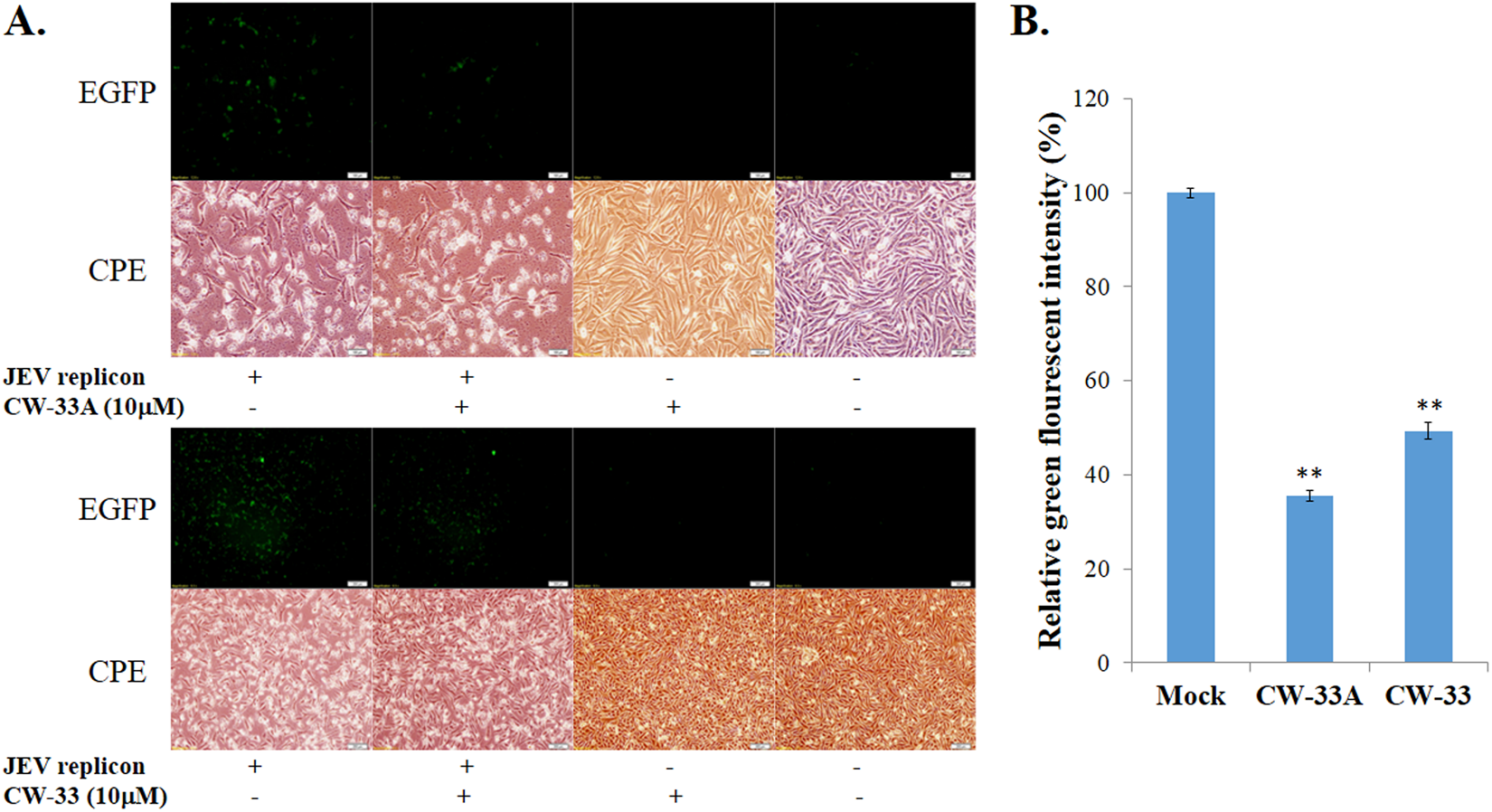
Inhibition of JEV replicon driven EGFP expression by CW-33A. TE-761 cells were transfected with or without JEV replicon and photographed 36 h post in the presence or absence of CW-33A (**A**, top) or CW-33 (**A**, bottom) for examining CPE by phase-contrast and detecting the EGFP expression using fluorescent microscopies. The relative green fluorescence intensity of EGFP in each group was measured using Image J software (**B**). **p value < 0.01 compared with mock treatment.

**Figure 9:**
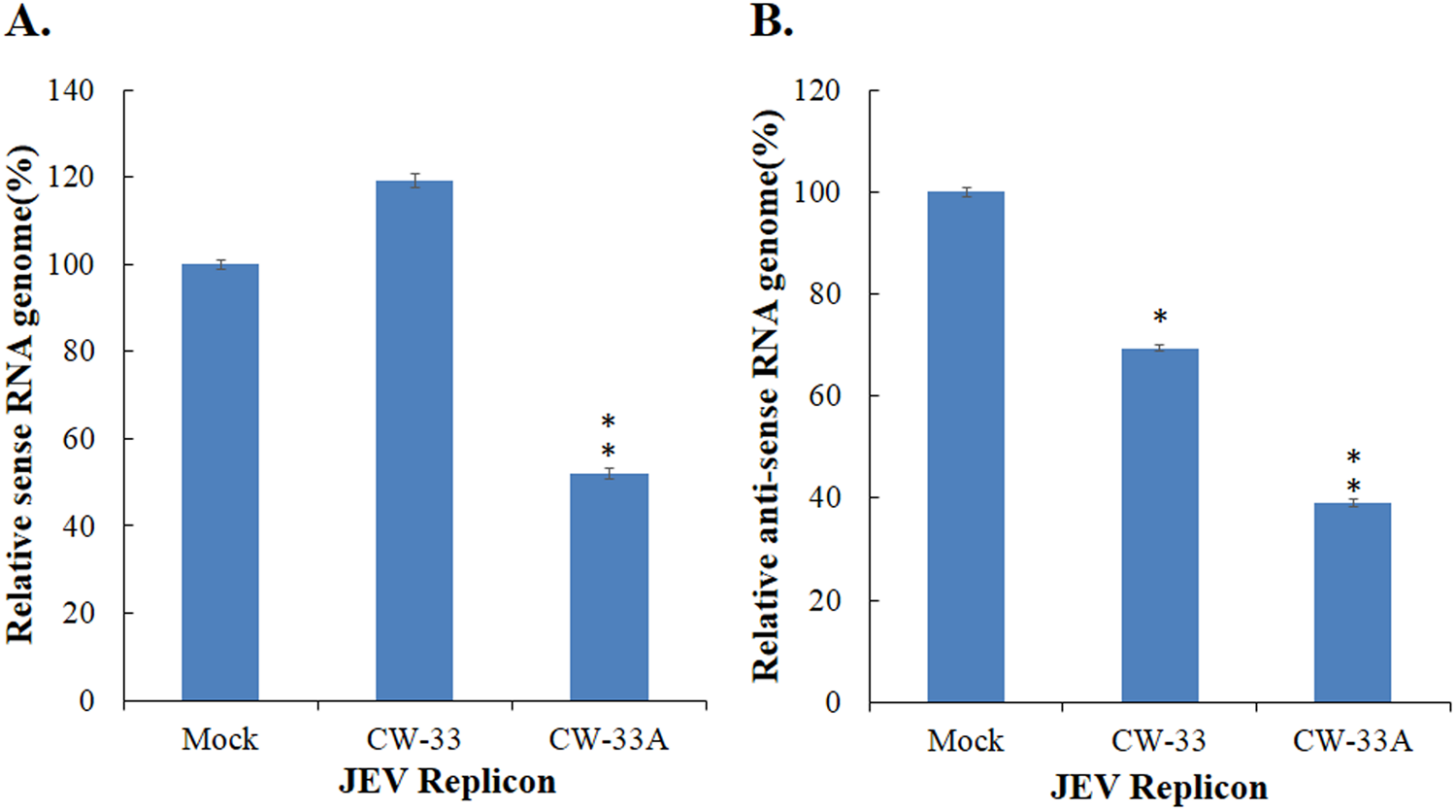
Inhibitory effect of CW-33A on the synthesis of viral RNA genome in DNA-launched JEV replicon-transfected cells. TE671 cells were transfected with the DNA-launched JEV replicon plasmid, and treated with CW-33 or CW-33A 1 h post transfection. Total RNAs from treated and transfected cells were extracted, and reverse transcripted with JEV specific primers. Relative viral positive- (**A**) and negative- (**B**) sense RNA genomes were measured by quantitative PCR and normalized by GAPDH mRNA. *p value < 0.05; **p value < 0.01. compared with mock-treated infected cells.

**Figure 10:**
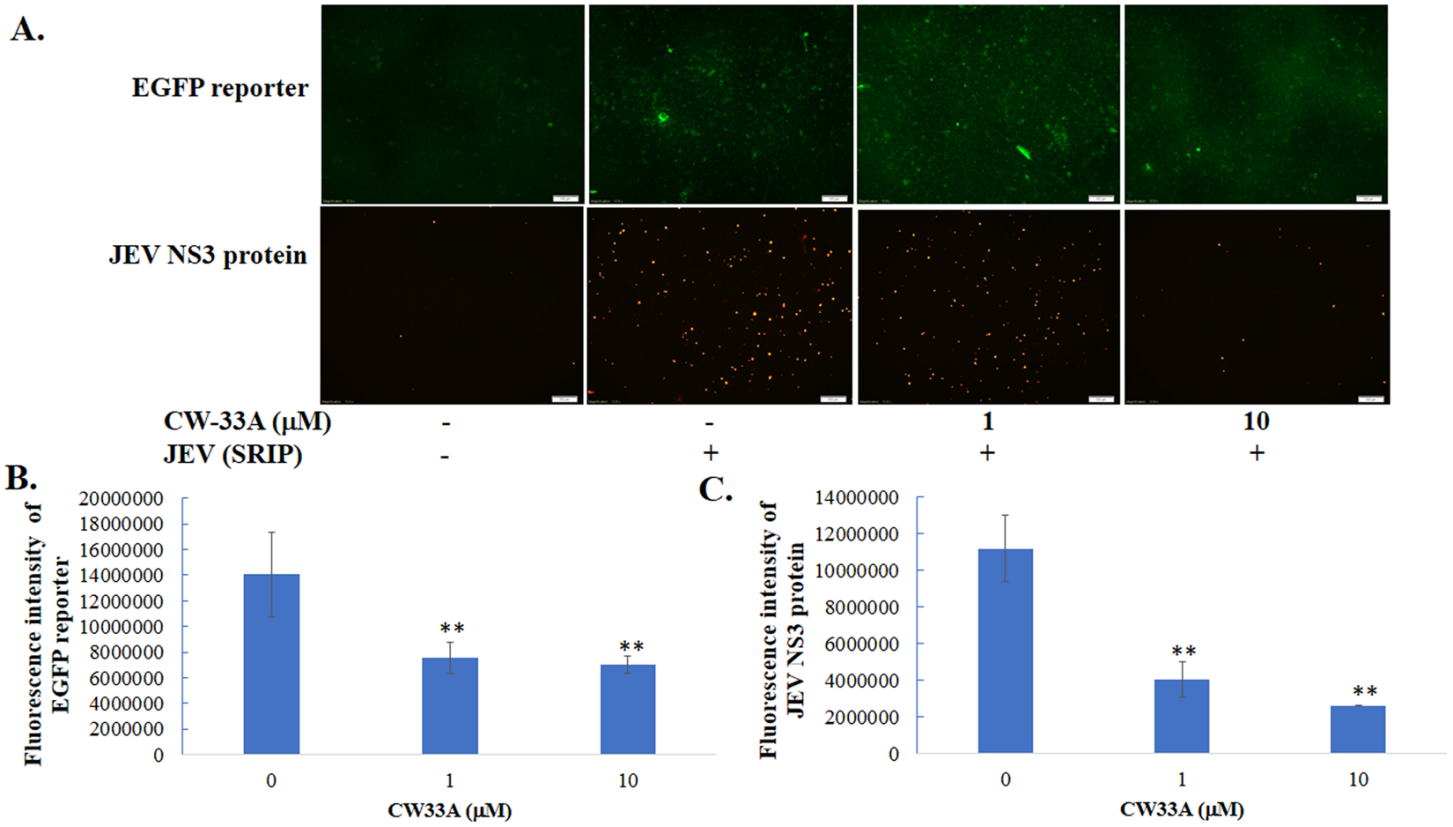
Inhibitory effect of CW-33A on the replication of JEV SRIPs. TE671 cells were infected JEV SRIP and immediately treated with CW-33A. After 72-h incubation, the green fluorescence images of SRIP-driven EGFP reporter in infected cells was taken using fluorescent microscopies (**A**). The infected cells were further performed using the immunofluorescent staining with anti-JEV NS-3 protein and Alexa Fluor 546-conjugated secondary antibodies; the red fluorescence images of SRIP-driven JEV NS3 protein in infected cells was photographed using fluorescent microscopies (**A**). Relative fluorescent intensity of SRIP-driven EGFP reporter and JEV NS3 protein was quantified using Image J, and shown in (**B**,**C**), respectively **p value < 0.01. compared with mock-treated infected cells.

CW-33 had no effect on the infectivity of virus particles and early entry into cells, but showed a significant inhibition on the late stage of JEV replication cycle^9^. Intracellular infectious virus assay demonstrated that CW-33A at 10 μM has a higher decrease in the intracellular production of JEV infectious particles than CW-33 24 or 48 hpi (Fig. 7). Moreover, a CMV promoter-launched JEV-EGFP replicon reported in our prior study^10^ was used to ascertain whether CW-33A influences on the viral protein expression and genome synthesis (Figs 8 and 9). Fluorescent microscopy revealed that CW-33A reduced the green fluorescence in the replicon-transfected cells and inhibited replicon-induced CPE in TE-671 cells (Fig. 8A), demonstrating CW-33A suppressing the synthesis of replicon-driven EGFP. Since negative-sense viral RNA genomes were wholly synthesized by viral RNA-dependent RNA polymerase-mediated replication complex in the CMV promoter-launched positive-sense RNA virus replicon system, relative levels of JEV positive- and negative-sense strand RNAs in replicon-transfected cells were further detected using real-time RT-PCR and normalized by housekeeping gene GAPDH (Fig. 9). CW-33A at 10 μM caused 48% and 61%, decreases of positive- and negative- sense synthesis, respectively. Meanwhile, CW-33A declined 25% in the ratio of negative-/positive-sense viral genome in JEV replicon-transfected cells compared to that in mock-treated replicon-transfected cells. JEV single-round infectious particles (SRIPs) containing an EGFP reported (named as JEV-EGFP SRIPs) were used to rule out the CMV promoter of DNA-launched replicon. CW-33A also significantly reduced the expression of EGFP reporter and JEV proteins in JEV SRIP-infected cells (Fig. 10). The results showed that antiviral activity of CW-33A against JEV was associated with the inhibitory action on viral protein expression and genomic RNA synthesis during the late stage of JEV replication in cells, in which linked with the intracellular JEV production.

## Discussion

CW-33 was previously identified as the lead compound exhibiting the anti-JEV activity *in vitro*^9^. On the basis of this promising activity, 6 mono substituted analogues with an fluoro, or methoxy group at the C-2, C-3, or C-4 anilino ring of CW-33 were evaluated for their anti-JEV activities included CPE inhibition and Sub-G1 reduction (Figs 2 and 3). When compared with the CPE inhibition activity of compound CW-33, CW-33B (3′- fluoroanilino substituent), CW-33C (4′- fluoroanilino substituent), CW-33E (3′-methoxyanilino substituent), and CW-33F (4′- methoxyanilino substituent) exhibited a markedly decrease of anti-JEV activities. Only CW-33A (2′- fluoroanilino substituent) and CW-33D (2′- methoxyanilino substituent) significantly reduced JEV-induced CPE in concentration-dependent manners. The result suggested that R3′ and R4′ in aniline ring play the crucial role in antiviral activity of CW-33 analogues, the modification on R2′ in aniline ring of CW-33 analogues appears as the main approach to improve the antiviral activity of CW-33. CW-33A placing an F atom as R2′ had higher anti-JEV activity than CW-33D placing anmethoxy group at 2′-position of aniline ring in CPE reduction assays. The phenomena could be responsible for the difference in water solubility and electronegativity of these analogues^11^.

CW-33A showed a higher inhibitory effect on infectivity during a 24-h period post infection, virus yield and plaque formation *in vitro* than CW-33 (Figs 4–6). CW-33A significantly inhibited the late stage of JEV replication *in vitro*, including intracellular virus production, viral protein expression and viral genome synthesis (Figs 7–10). The result indicated antiviral mechanism of CW-33A was consistent with that of CW-33 reported in a prior study that CW-33 declined the late stage of JEV replication via antagonizing the suppression of ERK1/2, Akt/mTOR, and Jak/STAT pathways by JEV^9^. CW-33A might have a clinical application on interfere post-entry stages during JEV replication.

Evaluating functional activities of CW-33 analogues signifies a fluoro atom at the C-2 anilino of CW-33 for improving the anti-JEV activity, displaying higher antiviral potential than CW-33. The structure-function relationship of CW-33 analogues might be applicable to develop novel agents against JEV infection.

## Materials and Methods

### Cells and viruses

Baby Hamster Kidney (BHK-21) and human medulloblastoma TE-671 cells were cultured in 5% fetal bovine serum (FBS)-containing MEM plus penicillin/streptomycin, glutamine, and pyruvate. BHK-21 cells were used to produce JEV T1P1 strain and determine viral titers by plaque assay. TE-671 cells were utilized for anti-JEV assays with CW-33 analogues.

### Synthesis of CW-33 analogues

Fourteen analogues of CW-33 (ethyl 2-(3′,5′-dimethylanilino)-4-oxo- 4,5-dihydrofuran- 3-carboxylate) were made according to the synthetic scheme in Fig. 1B. Sodium hydride (60%, 8.0 g, 0.2 mol), previously washed with dry n-hexane, was suspended in dry THF (40 ml) and added slowly, with stirring, over 10 min to a solution of diethyl malonate (32.0 g, 0.2 mol) in dry THF (60 ml). The reaction mixture was stirred in a water bath for 2 min, then cooled to 10–12 °C and chloroacetyl chloride (11.3 g, 0.1 mol) in dry THF (100 ml) was added dropwise over 10 min. The solution was kept at this temperature for 1 h, at 40–45 °C for another hour and various substituted aniline (12.1 g, 0.1 mol) in dry THF (100 ml) was then added dropwise over 20 min. The reaction mixture was left at room temperature overnight, heated under reflux for 2 h, then the THF solvent was evaporated off. The resulting residue was poured into ice water, and then extracted with chloroform. The organic layer was washed with water and dried with MgSO_4_. The solvent was partially evaporated and the concentrated residue refrigerated for 2 days. The precipitate was collected and recrystallized from ethanol to produce white crystals of target compounds 1–15 (Fig. 1C), also called as CW-33, CW-33A to CW-33F. The compounds were refined using high-performance liquid chromatography^9^; the identity and purity of CW-33 analogues were validated using ^1^H and ^13^C nuclear magnetic resonance (NMR) spectroscopy. The NMR spectra were listed in Supplemental Figs 1–12 and Table 1.

### MTT Cytotoxicity test

Cytotoxicity of CW-33 analogues to BHK-21 and TE-671 cells was tested using MTT assay, as described in our report^9^. 3 × 10^3^ cells/well were grown and treated with CW-33 analogue (1, 10, 100, 200, and 500 μM). After 48-h incubation, the cells were added with the MTT solution for 4 h reaction. Reduction of MTT to purple formazan dissolved by DMSO was measured by absorbance OD_570-630_ by an ELISA reader. Survival rate of treated cells in comparison with mock control cells was calculated as follows: Survival rate (%) = (*A*_treated cells_/*A*_control cells_) × 100%.

### Cytopathic effect inhibition, apoptosis reduction and infectivity inhibition assays

To investigate antiviral activity of CW-33 analogues against JEV, the inhibitory assays of viral CPE and virus-induced apoptosis were further executed. In the assay of CPE inhibition, TE-671 cells were co-treated with JEV (MOI of 0.05) and CW-33 analogues (10, 50, and 100 μM). Images of JEV-induced CPE were taken under microscope 36 h post treatment; apoptotic fractions of treated infected cells were determined using propidium iodide staining. After 15-min incubation, 10,000 cells from each sample are directly evaluated using flow cytometry with a 488 nm/620 nm. In the infectivity inhibition assay with immunofluorescence staining, JEV-infected cells with an MOI of 0.05 were fixed, permeabilized, and blocked after a 24-h incubation with the indicated concentrations of CW-33 and CW-33A, as described in our report^10^. Cells were stained with rabbit anti-JEV-NS3 (GeneTex, Inc) and secondary AF546 conjugated antibodies (ThermoFisher). Finally, the nucleus of total cells was stained DAPI (4′,6-diamidino-2-phenylindole). Infectivity was determined according to the ratio of NS3-positive cells to total cells stained with DAPI (4′,6-diamidino-2-phenylindole) by Image J software.

### Assays of virus yield reduction, intracellular viral particle, and plaque formation reduction

To measure the inhibitory activity of CW-33A on virus production, extracellular viruses (virus yield) and intracellular infectious particles (virions) of treated infected cells were quantitated using plaque assay. In virus yield reduction assay, BHK-21 cell monolayer in the 6-well plate was reacted with 100 μl of serial dilution of cultured media of infected cell harvested 36 h post treatment with CW-33A or CW-33. Cell monolayer was incubated for 1 h, overlaid with 2 ml 1.1% methylcellulose-containing MEM medium for 72 h, and then dyed using the naphthol blue-black solution. Extracellular infectious titer (JEV yield) was calculated based on the viral plaque forming units (pfu) per ml. In the quantitative assay of intracellular infectious particles, treated infected cells were harvested 24 or 48 hpi, and then performed through three freeze-thaw cycles. The lysate was diluted for measuring the intracellular infectious virus titer using plaque assay. In the plaque formation reduction assay, the BHK-21 cell monolayer was infected 100 pfu JEV particles and immediately treated with reacted with CW-33A or CW-33 for 1 h and, overlaid with 2 ml 1.1% methylcellulose-containing MEM medium, and then performed by the protocol of plaque assay.

### Viral RNA genome replication assays with JEV-EGFP replicon

A JEV-EGFP replicon, previously constructed in our laboratory^10^, was used to explore whether CW-33A influenced viral protein translation and RNA genome replication stages of JEV life cycle. TE-671 cells were transfected with JEV-EGFP replicon and simultaneously treated with 10 μM of CW-33A or CW-33. After 36 hours post treatment, the images of replicon-derived EGFP reporter in JEV-EGFP replicon-transfected cells were taken by fluorescent and optical microscopies. The fluorescent intensity of EGFP reporter in treated transfected cells was quantified by Image J. For detecting the synthesis of JEV sense and antisense genomes, total RNAs of replicon-transfected TE-671 cells treated with CW-33A or CW-33 were purified using PureLink Mini Total RNA Purification Kit (ThermoFisher), and performed using SYBR Green-based real time PCR with JEV-specific primer pairs, as described in our prior report [9]. The corresponding threshold cycle value(C_T_) for each sample was measured by 7300 Realtime PCR system (Applied Biosystems). Relative levels of JEV sense and antisense genomes were normalized by glyceraldehyde 3-phosphate dehydrogenase (GAPDH), and then quantitated.

### A combination assay of JEV single-round infectious particles (SRIPs) and immnunofluorescence

JEV single-round infectious particles (SRIPs) containing an EGFP reported (named as JEV-EGFP SRIPs) were generated as the following in our prior study^10^. TE-671 cells were infected with JEV-EGFP SRIPs at 10 TCID_50_ and soon treated with CW-33A. After 72-h incubation, the fluorescence images of SRIP-driven EGFP reporter in infected cells was taken using fluorescent microscopies, and relative green fluorescent intensity of EGFP reporter in treated infected cells was computed using Image J. Subsequently, treated infected cells were fixed, permeabilized, and blocked for immunofluorescence staining as described in our report^10^. SRIP-driven JEV NS3 protein in cells was discovered by rabbit anti-JEV-NS3 (GeneTex, Inc) and secondary AF546 conjugated antibodies (ThermoFisher). The fluorescent signals of JEV NS3 protein in treated infected cells was recorded using fluorescent microscopy and calculated by Image.

### Statistic analysis

All data of three independent experiments in each assay were collected and used to evaluate the statistical significance (*P* < 0.05) by Student’s *t*-test and Scheffe test.

## Electronic supplementary material

Supplementary Material
