# Supplementary material for: Structure analysis and antiviral activity of CW-33 analogues against Japanese encephalitis virus
Source: Sci Rep. 2018 Nov 9;8:16595. doi: 10.1038/s41598-018-34932-4 (PMC6226475; doi:10.1038/s41598-018-34932-4)
Supplement: Supplementary file 1 — Supplementary Material [file 41598_2018_34932_MOESM1_ESM.pdf]

SUPPLEMENTARY MATERIAL

Structure analysis and antiviral activity of CW-33 analogues against Japanese encephalitis virus

Jin-Cherng Lien[1 #] Ching-Ying Wang[2 #] Hsueh–Chou Lai[3,4 #] Chien-Yi Lu[2]

Yu-Fong Lin[2] Ging-Yan Gao[1] Kuan-Chung Chen[1] An-Cheng Huang[5]

Su-Hua Huang[6] Cheng-Wen Lin[2,6,7*]

[1]School of Pharmacy, China Medical University, Taichung, Taiwan

[2]Department of Medical Laboratory Science and Biotechnology, China Medical University, Taichung, Taiwan

[3]School of Chinese Medicine, China Medical University, Taichung, Taiwan

[4]Division of Hepato-gastroenterology, department of internal medicine, China Medical University Hospital, Taichung, Taiwan

[5]Department of Nursing, St. Mary's Junior College of Medicine, Nursing and Management, Yilan County, Taiwan

[6]Department of Biotechnology, Asia University, Wufeng, Taichung, Taiwan

[7]Chinese Medicine Research center, China Medical University, Taichung, Taiwan

[#] co-first author

*Corresponding author: Cheng-Wen Lin, PhD, Professor. Department of Medical Laboratory Science and Biotechnology, China Medical University; 91 Hsueh-Shih Road, Taichung 404, Taiwan

Fax: 886-4-2205-7414.

Email: cwlin@mail.cmu.edu.tw

Supplemental Figure Legends

Supplemental Figure 1. 1H NMR ($CDCl_3$, 200 MHz) for compound CW-33A
Supplemental Figure 2. 13C NMR ($CDCl_3$, 50 MHz) for compound CW-33A
Supplemental Figure 3. 1H NMR ($CDCl_3$, 400 MHz) for compound CW-33B
Supplemental Figure 4. 13C NMR ($CDCl_3$, 100 MHz) for compound CW-33B
Supplemental Figure 5. 1H NMR ($CDCl_3$, 500 MHz) for compound CW-33C
Supplemental Figure 6. 13C NMR ($CDCl_3$, 125 MHz) for compound CW-33C
Supplemental Figure 7. 1H NMR ($CDCl_3$, 50 MHz) for compound CW-33D
Supplemental Figure 8. 13C NMR ($CDCl_3$, 50 MHz) for compound CW-33D
Supplemental Figure 9. 1H NMR ($CDCl_3$, 200 MHz) for compound CW-33E
Supplemental Figure 10. 13C NMR ($CDCl_3$, 50 MHz) for compound CW-33E
Supplemental Figure 11. 1H NMR ($CDCl_3$, 200 MHz) for compound CW-33F
Supplemental Figure 12. 13C NMR ($CDCl_3$, 50 MHz) for compound CW-33F
Supplemental Figure 13. Histograms for cell cycle analysis of JEV-infected cells treated with the indicated compound using flow cytometry assay with propidium iodide staining. A, CW-33; B, CW-33A; C, CW-33B, D, CW-33C; E, CW-33D; F, CW-33E; G, CW-33F.

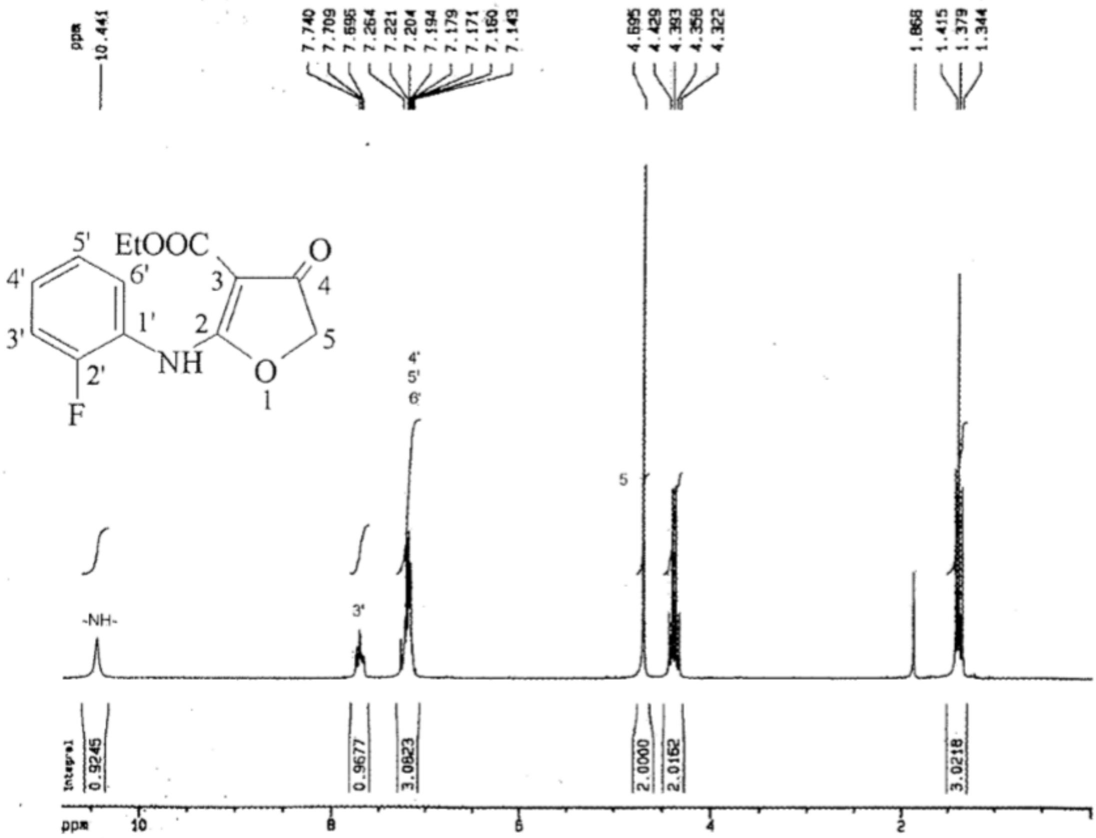

Supplemental Figure 1. 1H NMR ($CDCl_3$, 200 MHz) for compound CW-33A

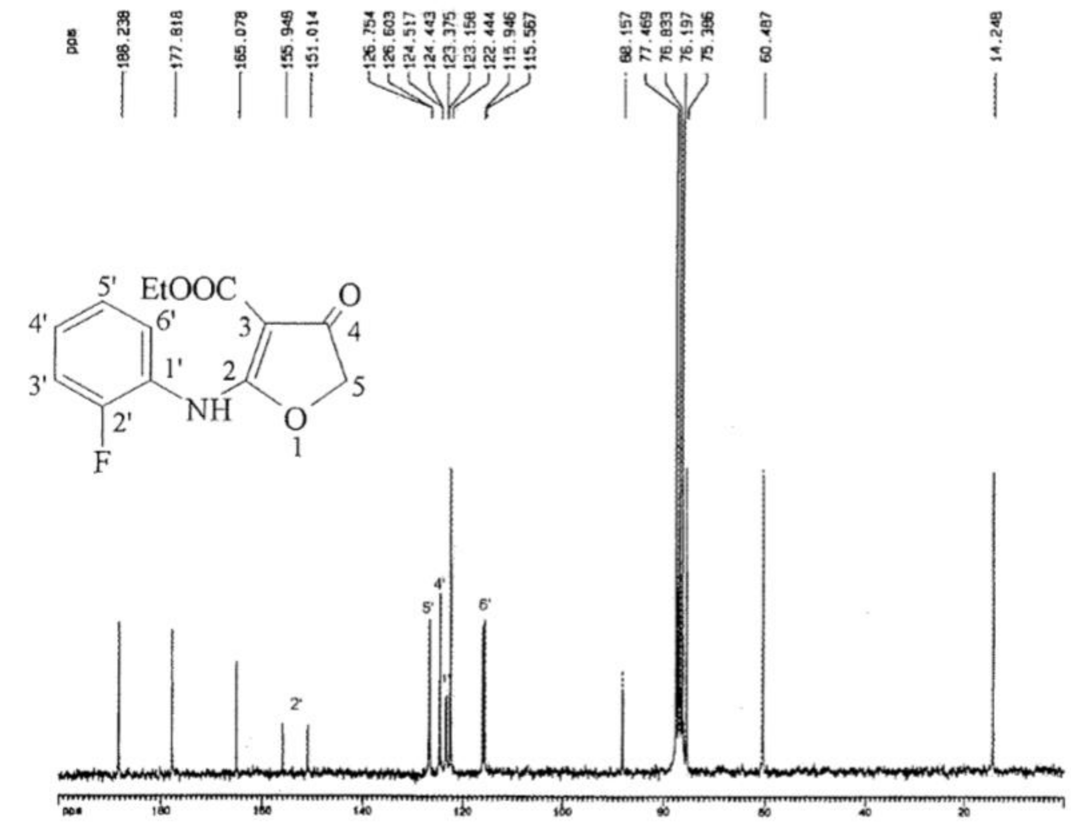

Supplemental Figure 2. 13C NMR ($CDCl_3$, 50 MHz) for compound CW-33A

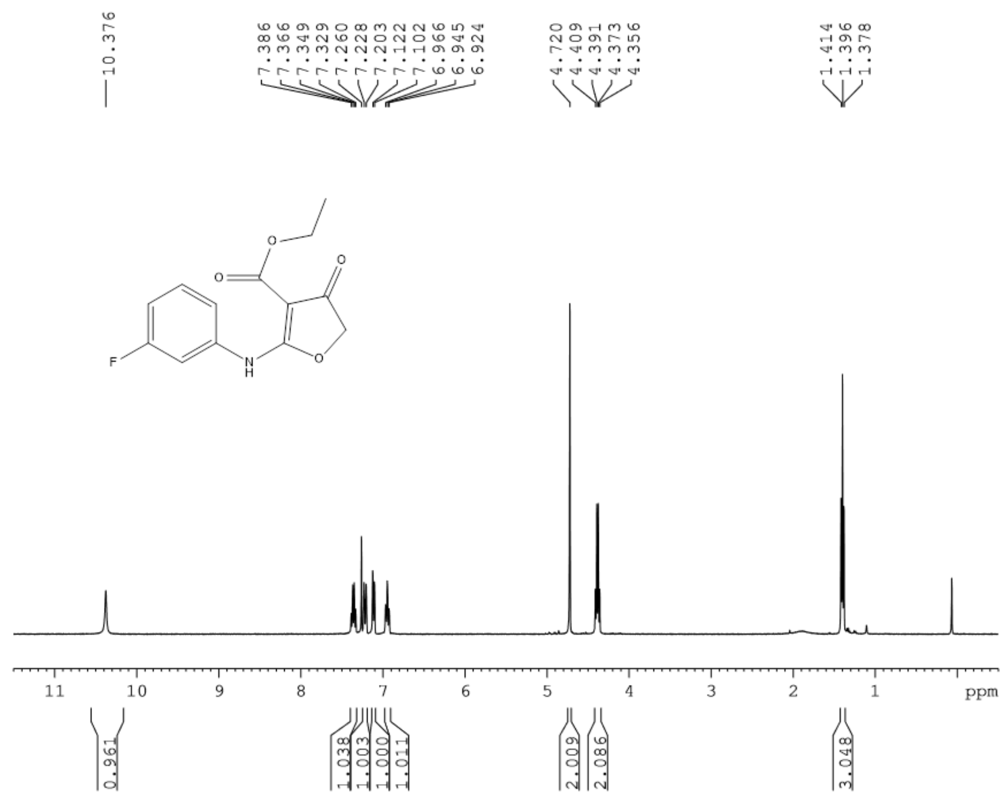

Supplemental Figure 3. 1H NMR ($CDCl_3$, 400 MHz) for compound CW-33B

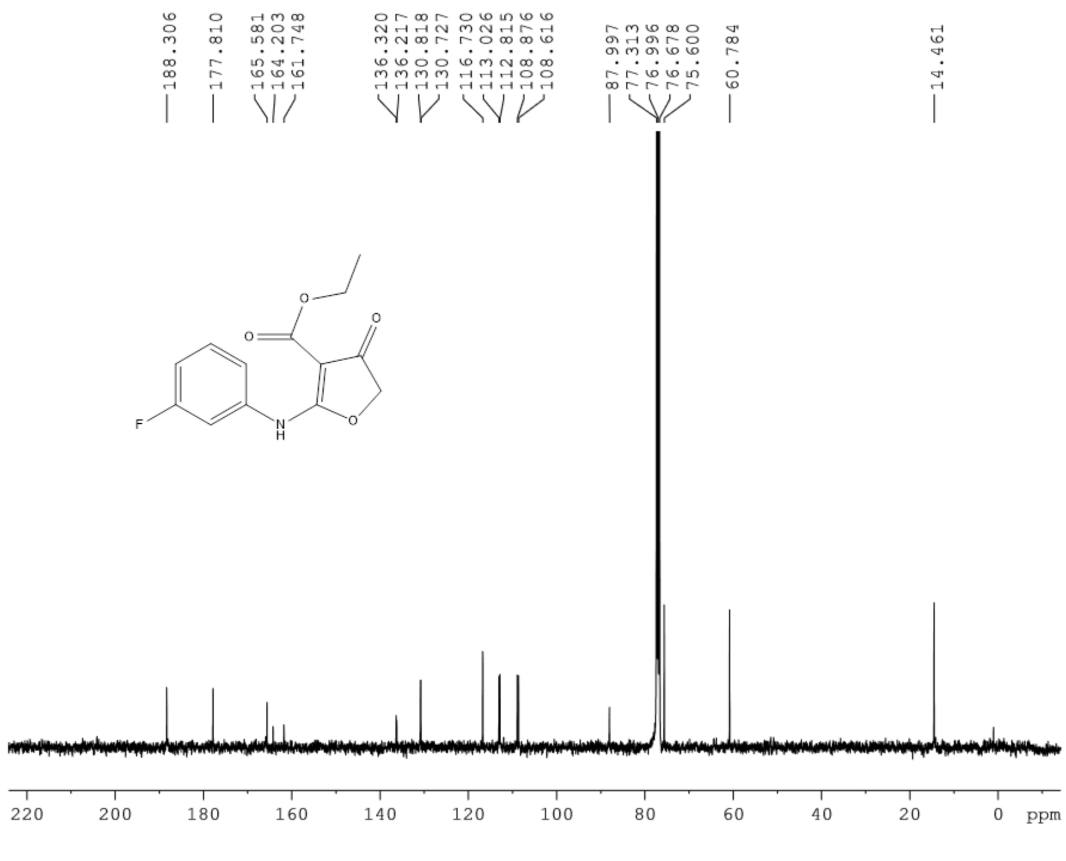

Supplemental Figure 4. 13C NMR ($CDCl_3$, 100 MHz) for compound CW-33B

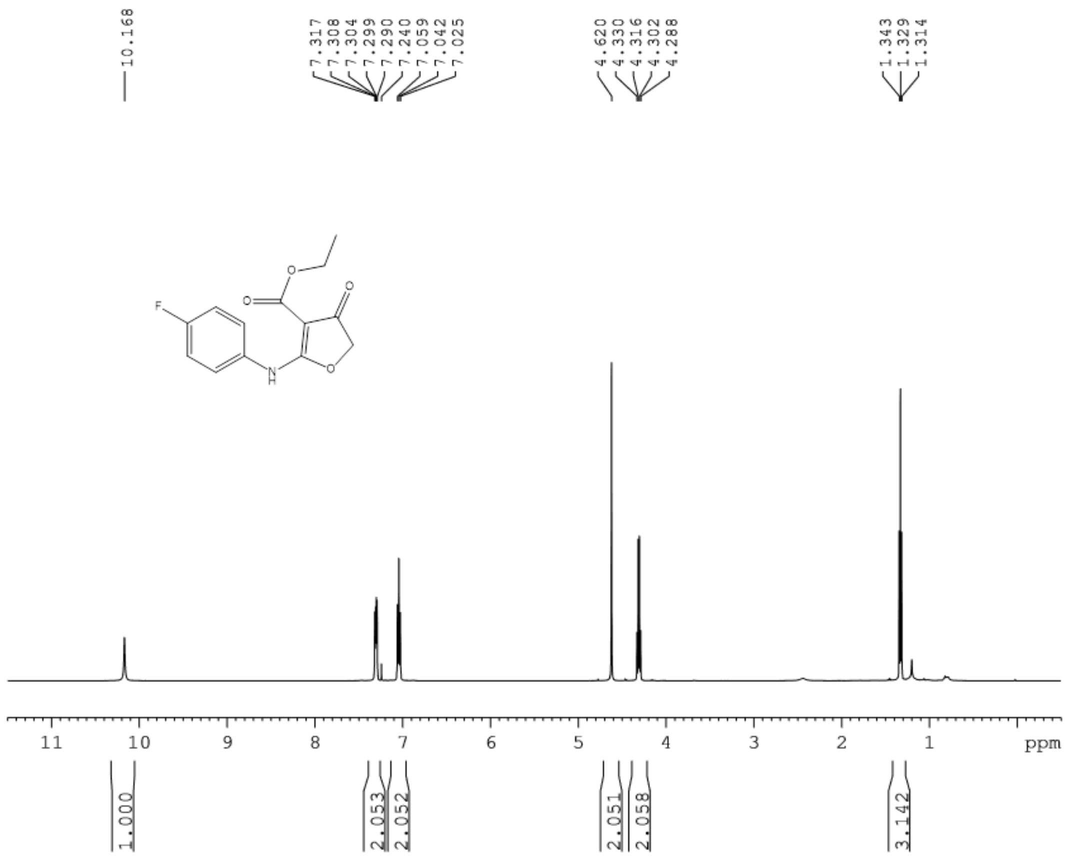

Supplemental Figure 5. 1H NMR ($CDCl_3$, 500 MHz) for compound CW-33C

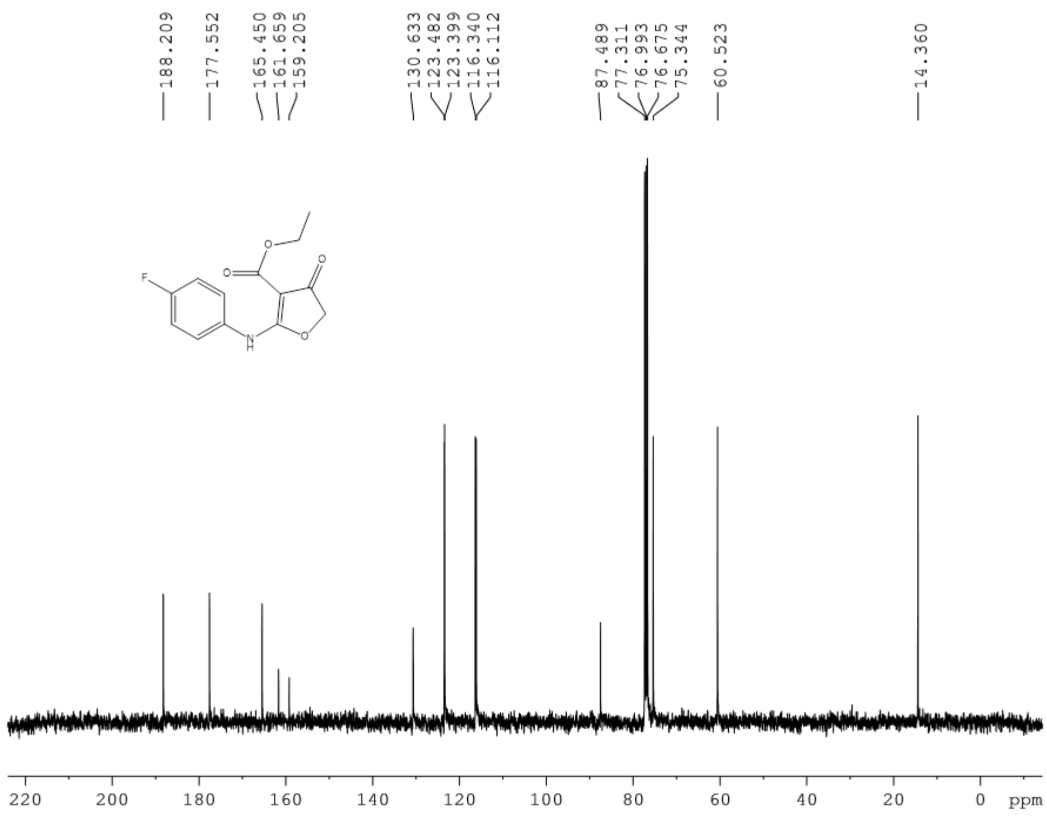

Supplemental Figure 6. 13C NMR ($CDCl_3$, 125 MHz) for compound CW-33C

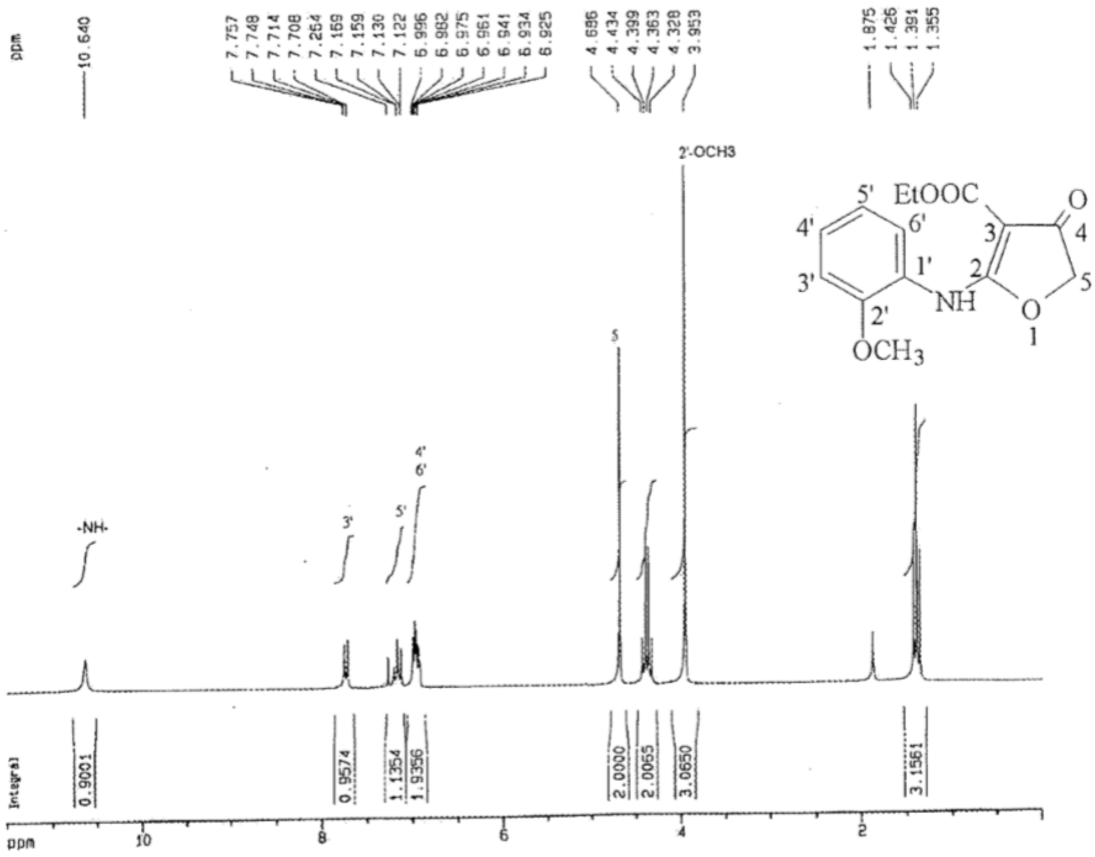

Supplemental Figure 7. 1H NMR ($CDCl_3$, 50 MHz) for compound CW-33D

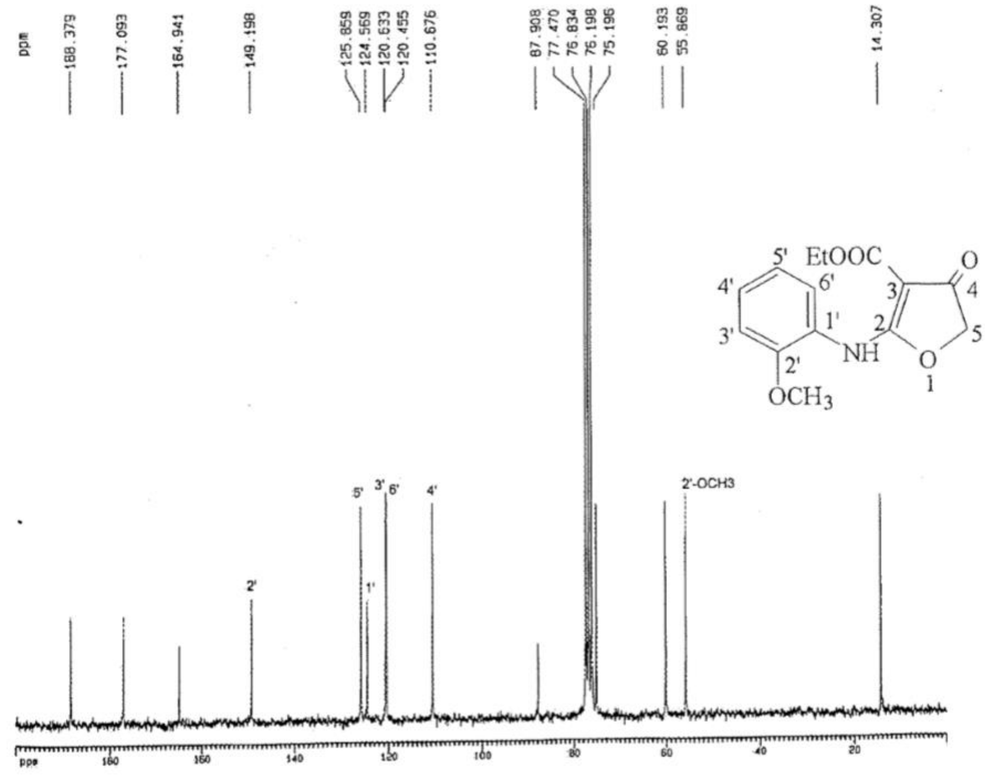

Supplemental Figure 8. 13C NMR ($CDCl_3$, 50 MHz) for compound CW-33D

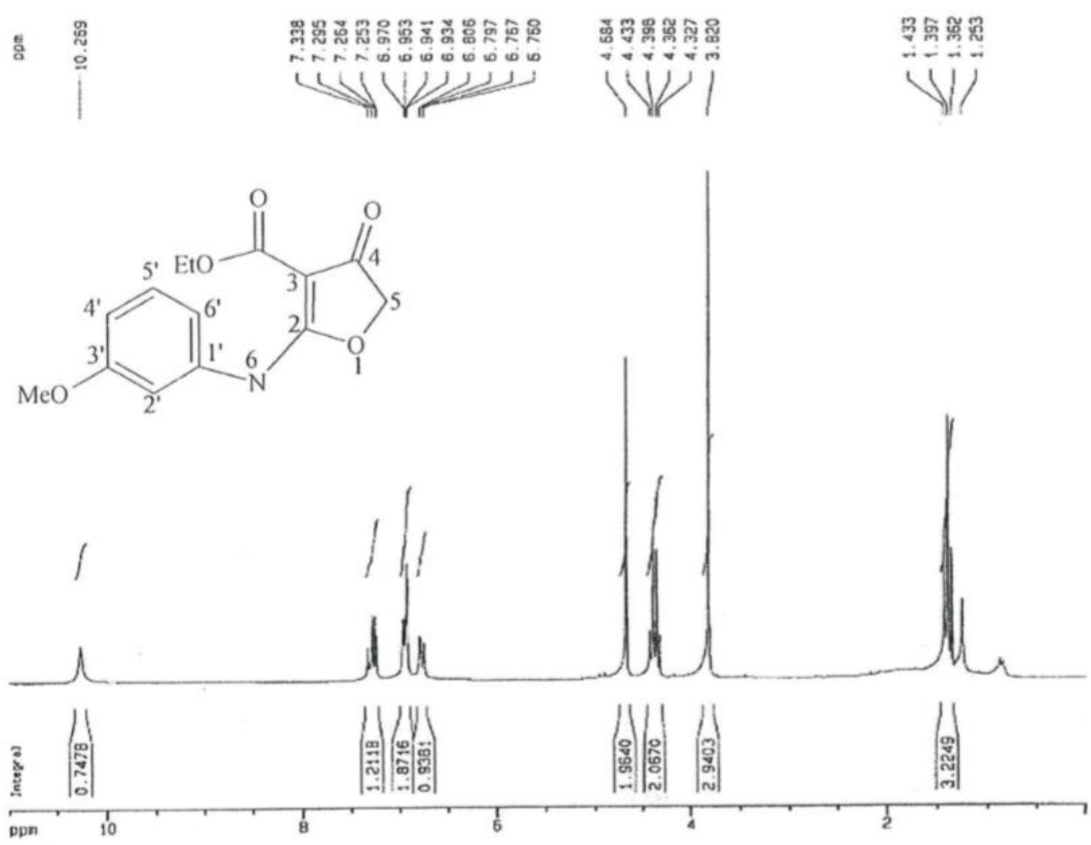

Supplemental Figure 9. 1H NMR ($CDCl_3$, 200 MHz) for compound CW-33E

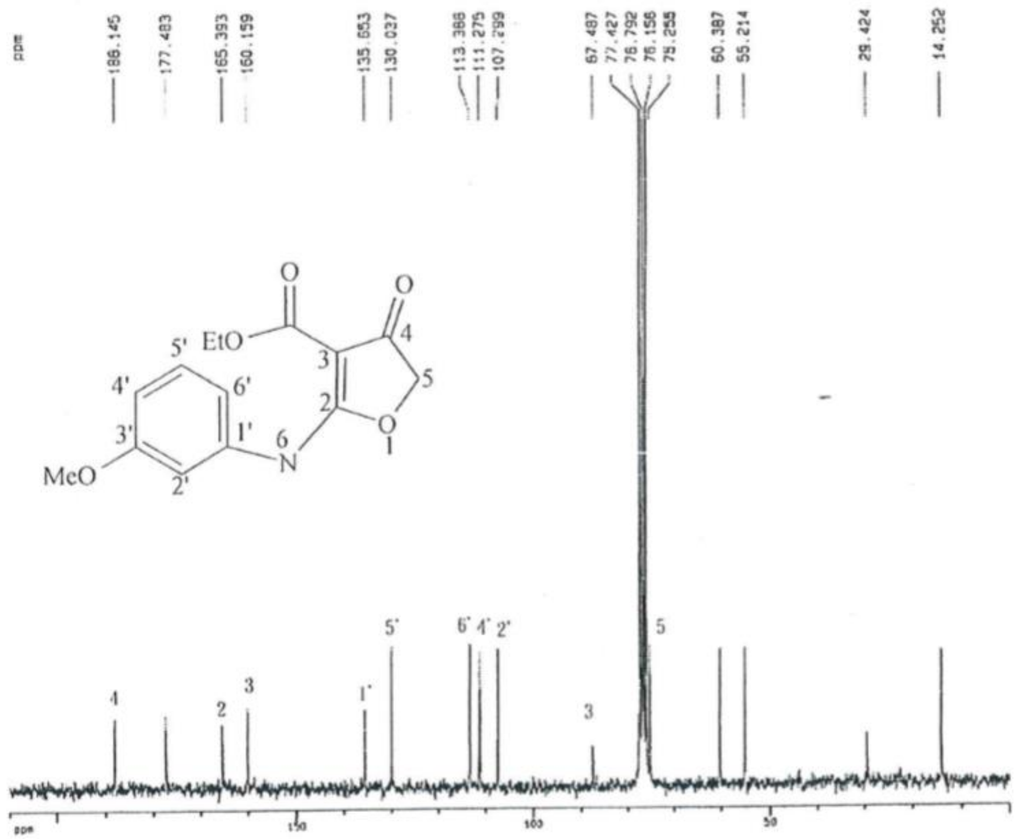

Supplemental Figure 10. 13C NMR ($CDCl_3$, 50 MHz) for compound CW-33E

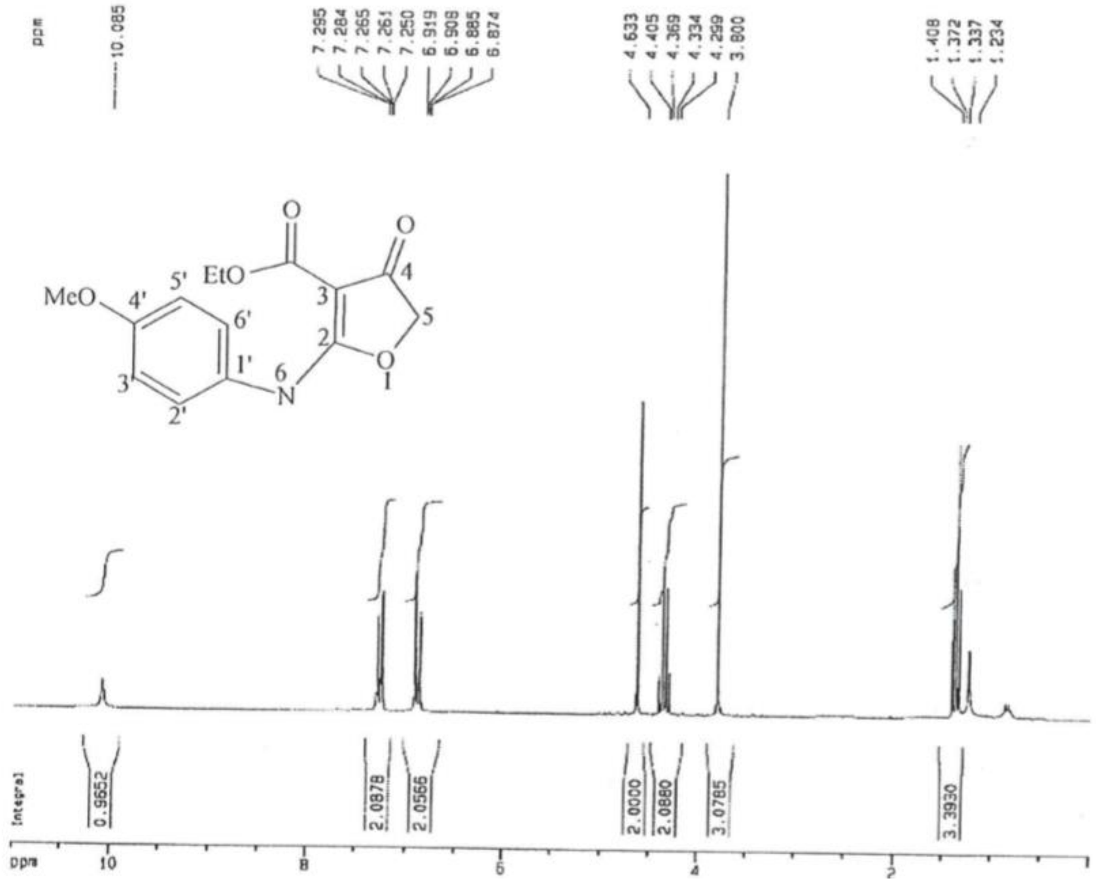

Supplemental Figure 11. 1H NMR ($CDCl_3$, 200 MHz) for compound CW-33F

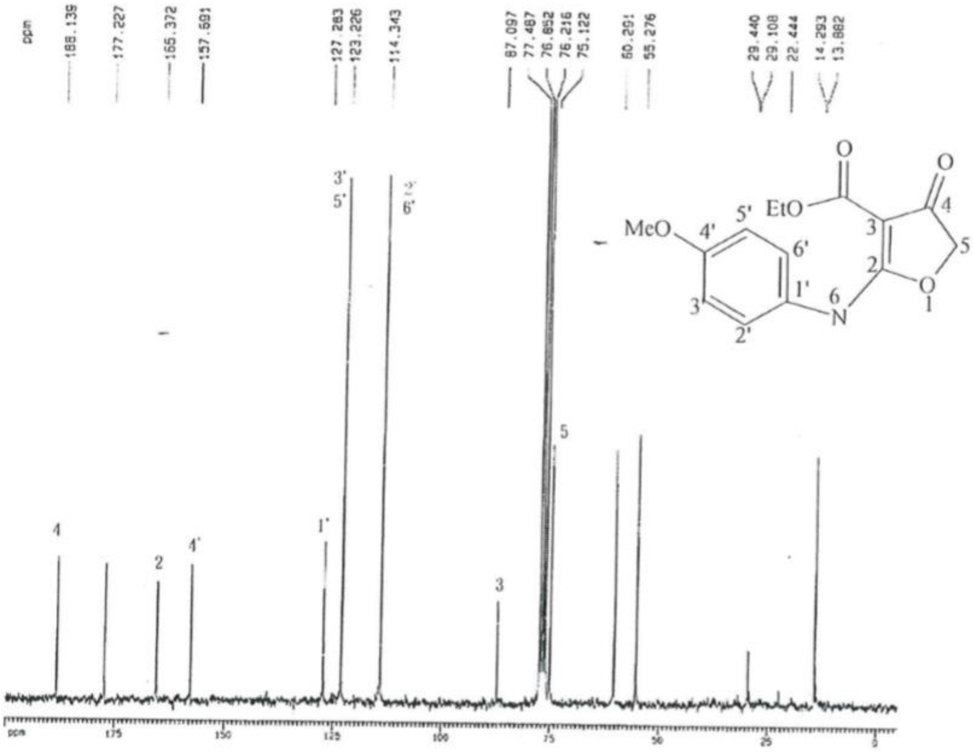

Supplemental Figure 12. 13C NMR ($CDCl_3$, 50 MHz) for compound CW-33F

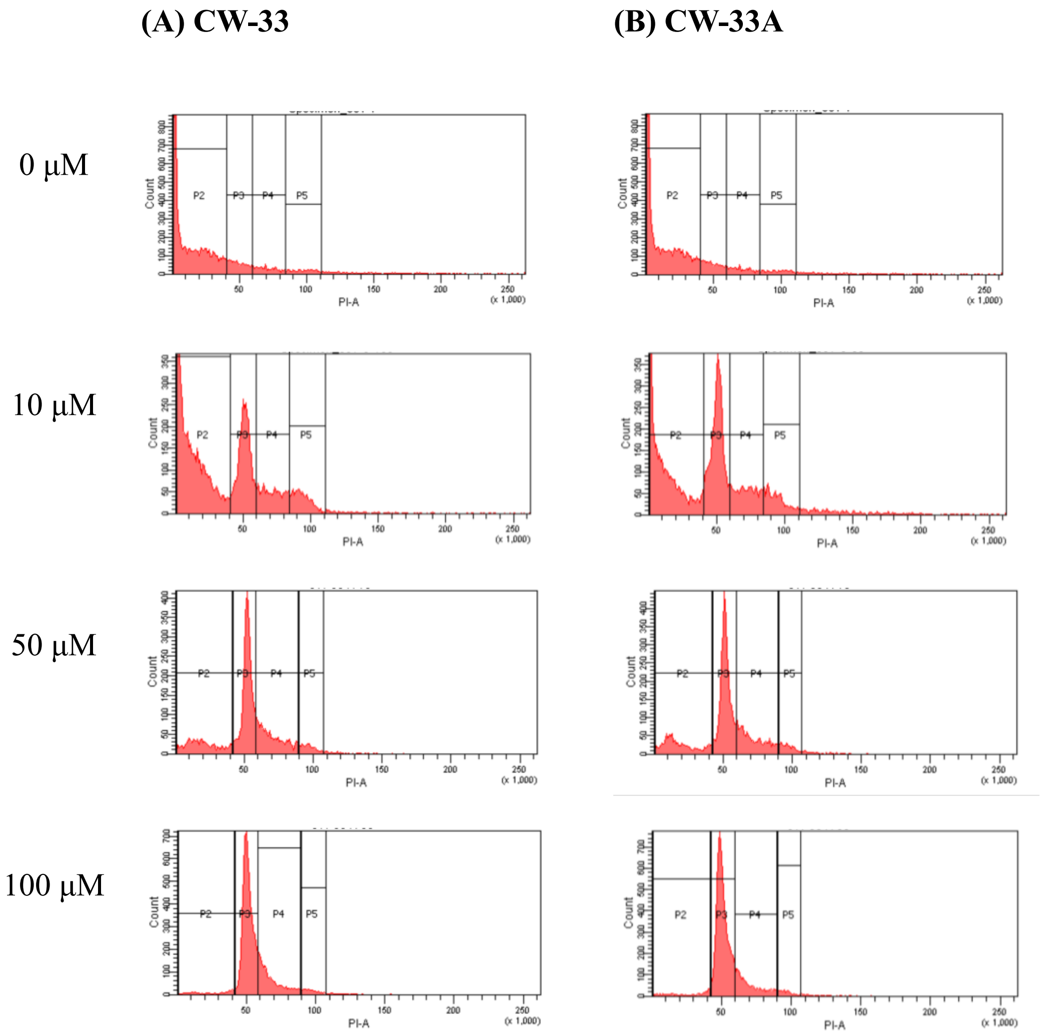
(A) CW-33
(B) CW-33A
0 µM
10 µM
50 µM
100 µM
Count
PI-A
P2
P3
P4
P5
(x 1,000)

(C) CW-33B **(D) CW-33C**

0 μM

10 μM

50 μM

100 μM

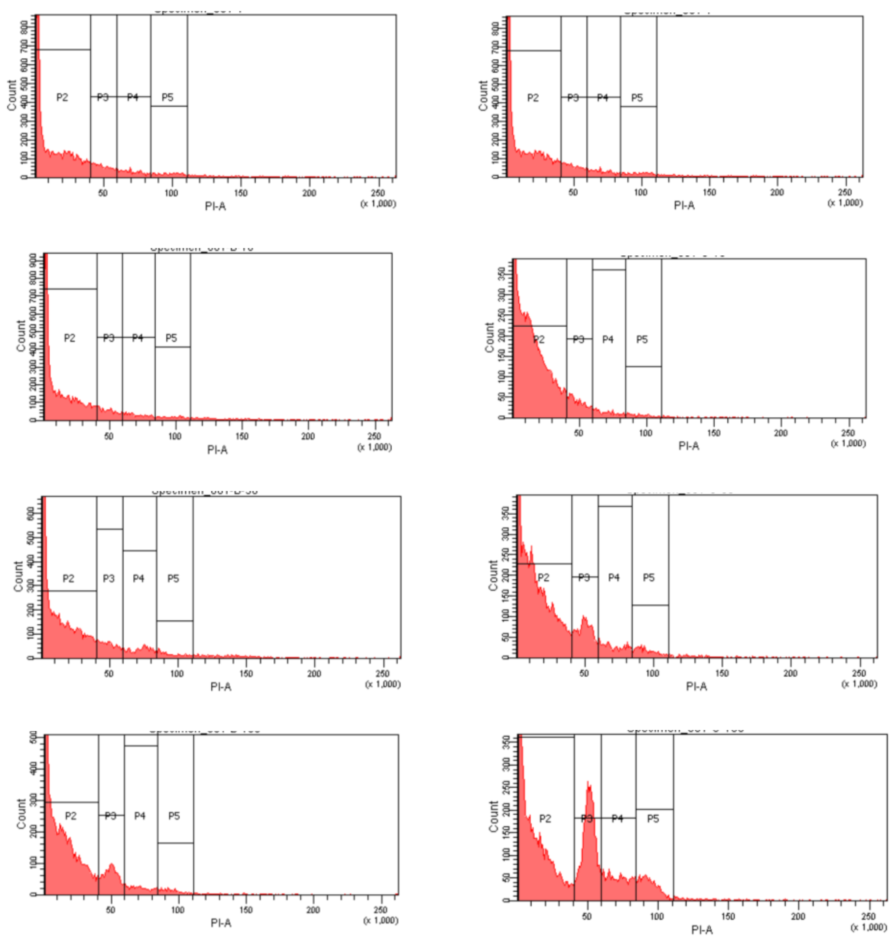

(E) CW-33D | (F) CW-33E

0 µM

10 µM

50 µM

100 µM

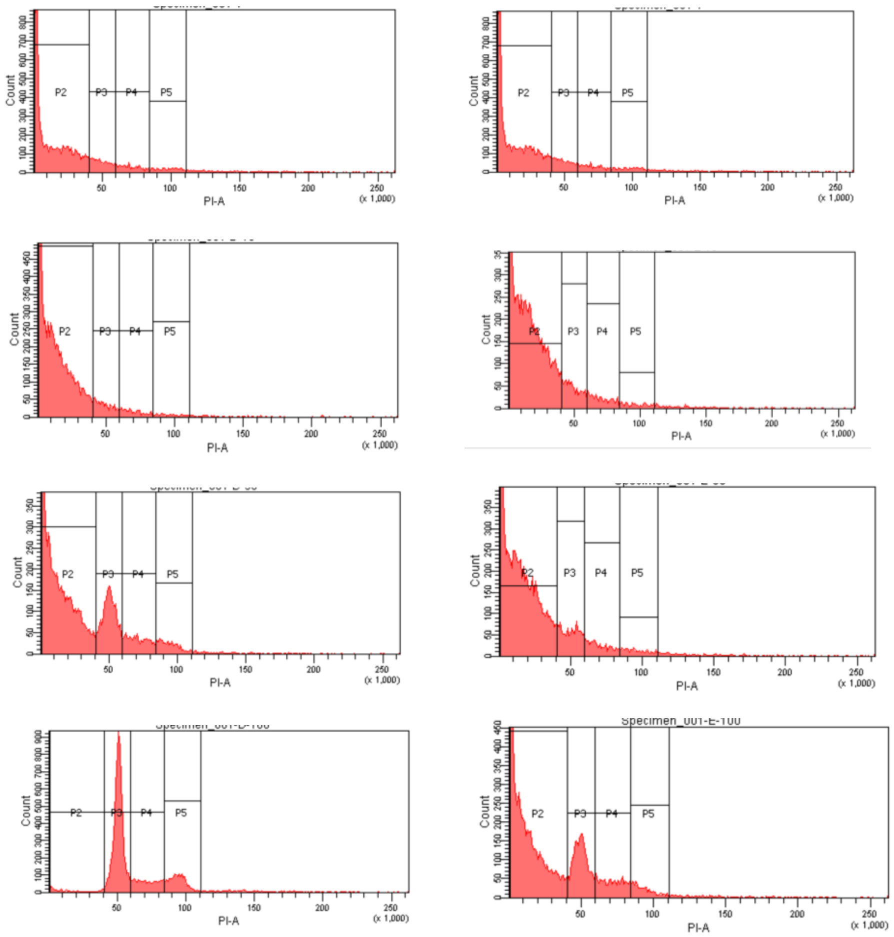

(G) CW-33F

0 µM

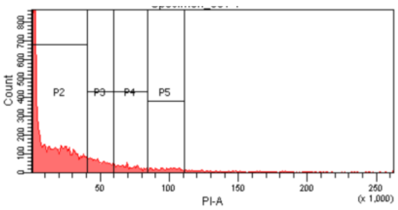

10 µM

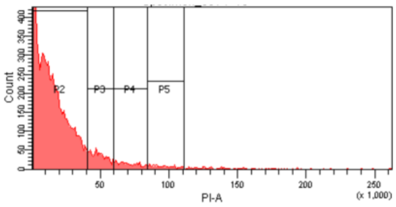

50 µM

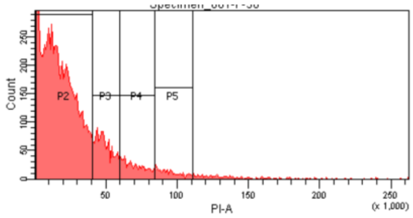

100 µM

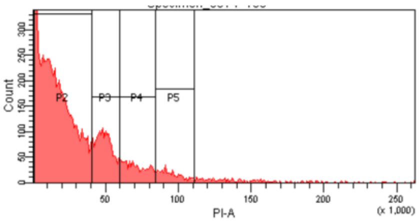

Supplemental Figure 13. Histograms for cell cycle analysis of JEV-infected cells treated with the indicated compound using flow cytometry assay with propidium iodide staining. A, CW-33; B, CW-33A; C, CW-33B, D, CW-33C; E, CW-33D; F, CW-33E; G, CW-33F.
